# Multi-Body Interactions in Molecular Docking Program Devised with Key Water Molecules in Protein Binding Sites

**DOI:** 10.3390/molecules23092321

**Published:** 2018-09-11

**Authors:** Wei Xiao, Disha Wang, Zihao Shen, Shiliang Li, Honglin Li

**Affiliations:** 1 School of Information Science and Engineering, East China University of Science and Technology, 130 Meilong Road, Shanghai 200237, China; xiaowei1012163@163.com; 2 Shanghai Key Laboratory of New Drug Design, School of Pharmacy, East China University of Science and Technology, 130 Meilong Road, Shanghai 200237, China; dishawang@foxmail.com (D.W.); saika_mx@163.com (Z.S.); slli403@163.com (S.L.)

**Keywords:** water molecules, multi-body docking, multi-objective optimization, optimization variables

## Abstract

Water molecules play an important role in modeling protein-ligand interactions. However, traditional molecular docking methods often ignore the impact of the water molecules by removing them without any analysis or keeping them as a static part of the proteins or the ligands. Hence, the accuracy of the docking simulations will inevitably be damaged. Here, we introduce a multi-body docking program which incorporates the fixed or the variable number of the key water molecules in protein-ligand docking simulations. The program employed NSGA II, a multi-objective optimization algorithm, to identify the binding poses of the ligand and the key water molecules for a protein. To this end, a force-field-based hydration-specific scoring function was designed to favor estimate the binding affinity considering the key water molecules. The program was evaluated in aspects of the docking accuracy, cross-docking accuracy, and screening efficiency. When the numbers of the key water molecules were treated as fixed-length optimization variables, the docking accuracy of the multi-body docking program achieved a success rate of 80.58% for the best RMSD values for the recruit of the ligands smaller than 2.0 Å. The cross-docking accuracy was investigated on the presence and absence of the key water molecules by four protein targets. The screening efficiency was assessed against those protein targets. Results indicated that the proposed multi-body docking program was with good performance compared with the other programs. On the other side, when the numbers of the key water molecules were treated as variable-length optimization variables, the program obtained comparative performance under the same three evaluation criterions. These results indicated that the multi-body docking with the variable numbers of the water molecules was also efficient. Above all, the multi-body docking program developed in this study was capable of dealing with the problem of the water molecules that explicitly participating in protein-ligand binding.

## 1. Introduction

Protein-ligand docking simulation plays a key role in the general field of molecular docking, because of the effect of the discovery of lead compounds and the analysis of structure-activity relationships. Molecular docking methods mainly consist of sampling of the conformational space and scoring of the resultant structures. Sampling typically includes ligand conformation, protein conformation, and ligand position with respect to the protein. While scoring seeks to distinguish optimal binding poses by estimating binding affinity. Although the current sampling and scoring algorithms are often able to predict the optimal binding pose [1] and achieve a satisfactory prediction of the binding affinity [2], one remaining challenge in molecular docking is positioning the interface water molecules and then evaluating the energetic contribution implied by the presence or displacement of the water molecules in the binding sites of crystal structures. 

Generally, water molecules found in crystal structures contribute to the shape and the flexibility of the binding sites, mainly by mediating the formation of the hydrogen bonds between the proteins and their ligands [3]. However, the impact of the water molecules is often ignored directly in traditional molecular docking simulations. Only occasionally may one or two catalytic water molecules be retained for the design of enzyme inhibitors, based on further experimental structural or kinetic data [4]. But so far, several protein-ligand docking studies have been performed to elucidate that the presence of the water molecules in the binding sites of crystal structures plays an important role in protein-ligand recognition [5,6,7,8]. Hussain et al. [9] found that the incorporation of explicit water molecules in the binding site of actin enabled improved accuracy for ligands with the formamide moiety in quantitative structure-activity relationship (QSAR) modeling. Huang et al. [7] used explicit water sites in 24 proteins to improve their docking enrichment factors and reduce false positives. Verdonk et al. [10] used crystallographic waters to improve docking performance by up to 20%. In addition, the cross-docking simulations had been performed on a number of ligand-protein complexes for various proteins whose crystal structures contain water molecules in their binding sites. And a statistically significant overall increase in accuracy was observed when water molecules were included [11]. Consequently, it is reasonable to consider the water molecules as an effective strategy in molecular docking. 

In fact, various investigations [4,12,13] and methods [14,15,16,17] have been developed to deal with the problem of the protein-ligand docking with water molecules. One simple way is to include the water molecules as a static part of the protein structures in the docking simulations. This strategy is feasible only if the number of the key water molecules is few. Then it will sum up to 2^n^ separate docking programs in parallel when n water molecules are assumed to take part in the protein-ligand docking [18]. Another way is to include the water molecules as a static part of the ligands. For instance, AutoDock [19] introduced a force field and hydration docking method that enabled the automated prediction of waters mediating the binding of ligands with target proteins. And the hydration force field accounted for the entropic and enthalpic contributions of discrete waters to ligand binding, improving energy estimation accuracy and docking performance. Additionally, the effects of displacing, neglecting and targeting water molecules in drug design led to some simple empirical rules about which chemical groups are most suitable to displace tightly-bound water molecules [20]. Thermodynamic integration methods had been used to describe in detail the changes in free energy of binding upon substitutions made to ligands aimed at displacing tightly-bound water molecules to improve binding affinity [21]. Furthermore, Bettens et al. [22,23] had succeeded in predicting the interactions of water molecules in the multi-body interactions. Despite a variety of scoring functions developed by various research groups, most of these do not incorporate the numbers of the water molecules alone as the optimization variables to form the multi-body docking of protein-water-ligand. 

Motivated by the above discussions, in this study, a multi-body docking program which incorporates the key water molecules in protein-ligand docking simulations was introduced. Especially, the numbers of the key water molecules were respectively treated as fixed-length and variable-length optimization variables in the model of the multi-body interactions. The program employed NSGA II, a multi-objective optimization algorithm, to identify the binding poses of the ligand and the key water molecules for a protein. And a force-field-based hydration-specific scoring function was designed to favor the estimating of the binding affinity. In addition, the performance of the multi-body docking program was evaluated in aspects of the docking accuracy, cross-docking accuracy, and screening efficiency.

## 2. Results and Discussion

### 2.1. Results of Multi-Body Docking Considering the Key Water Molecules as Fixed-Length Optimization Variables

**Figure 1:**
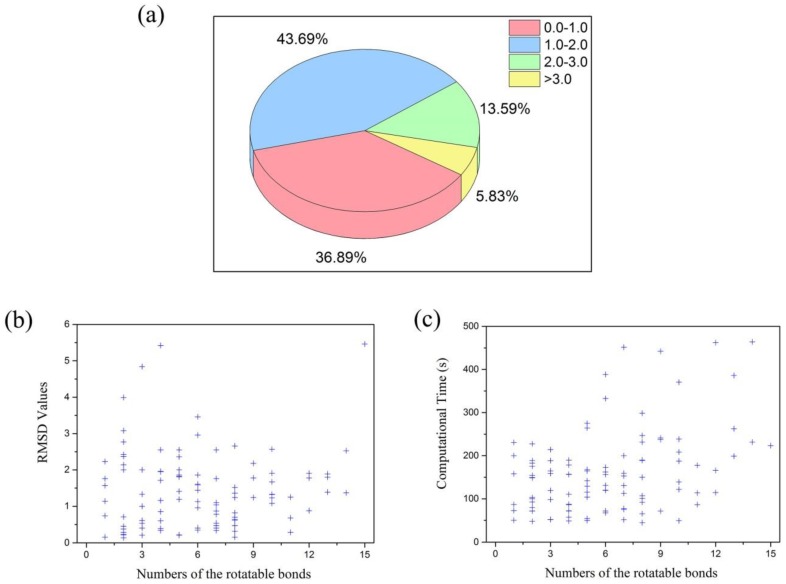
(**a**) The best RMSD values for the recruit of the ligands in those datasets. (**b**) The relationship between the numbers of the rotatable bonds and the best RMSD values for the recruit of the ligands. (**c**) The relationship between the numbers of the rotatable bonds of the ligands and the computational time.

Generally, the docking accuracy is based on the root-mean-square deviation (RMSD) value of the locations of all heavy atoms of the ligand in the docked pose from those of the crystal structure. It is acceptable if the RMSD value of the ligand is smaller than 2.0 Å. As mentioned above in the preparation of the data sets, a test set of 103 hydration crystal structures was used to evaluate the docking accuracy of the multi-body docking considering the key water molecules as fixed-length optimization variables. The results (Figure 1a) showed that 80.58% of the best RMSD values for the recruit of the ligands were smaller than 2.0 Å, that is, 80.58% of the crystal structures obtained accurate binding poses after the optimizations (The detail results are shown in Table S3). 

Additionally, as can be seen from the relationship between the best RMSD values and the flexibility of the ligands in Table 1 and Figure 1b, most of the best RMSD values for the recruit of the ligands focused on the regions of one to ten rotatable bonds of the ligands. And the best RMSD values of less than 2.0 Å mainly focused on the regions of two to six rotatable bonds of the ligands. In addition, most of the computational time that was lower than 200 s focused on the regions of one to eight rotatable bonds of the ligands (Figure 1c). Furthermore, their average time increased with the numbers of the rotatable bonds of the ligands (Table 1). 

**Figure 2:**
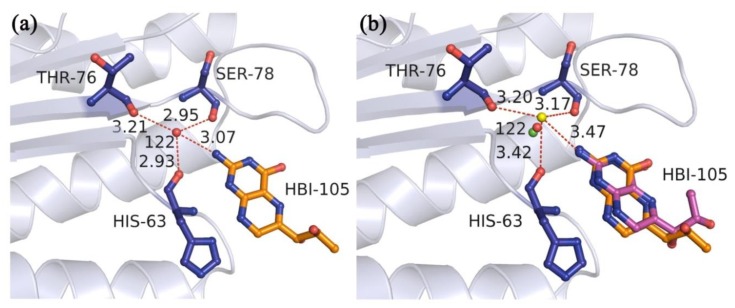
(**a**) The hydrogen bond interactions of the experiment-determined water molecule W122 in the binding site of crystal structure 1DCP. (**b**) The docked pose of the best RMSD value for the recruit of the ligand in the multi-body docking simulation of crystal structure 1DCP. The ligands in the X-ray pose and the docked pose are shown as orange and magenta ball-and-stick models, respectively. The W122, the original site of the water molecule, and the optimized water molecule in the multi-body docking simulation are represented as red, green and yellow spheres, respectively. The hydrogen bonds between the water molecules and the nearby amino acid residues (blue ball-and-stick model) or the ligands (orange ball-and-stick model) are represented by red dashed lines. Numbers beside the dashed lines are the lengths.

By way of illustration, let us take 1DCP (the crystal structure of DCOH, a bifunctional protein-binding transcriptional coactivator, complexed with biopterin) as an example. In crystal structure 1DCP (Figure 2a), the experiment-determined water molecule W122 (red sphere) played a significant role in simultaneously interacting with the nearby three residues HIS-63, THR-76, and SER-78 as well as the ligand atom. Thus, the W122 was treated as the key water molecule in the binding site. The original site of the optimized water molecule (green sphere) in the docking simulation was 0.54 Å away from W122. As the original sit was close to the experiment site of the water molecule, the optimization cost could be greatly reduced. After the optimizations, the best RMSD value for the recruit of the ligand in the multi-body docking simulations was 1.33 Å (Figure 2b), and only the rotatable bond of the ligand far away from the key water molecule had a small rotation. Additionally, the optimized water molecule (yellow sphere) was 1.21 Å away from W122. And it had the hydrogen bond interaction with the oxygen atoms of the same three residues HIS-63, THR-76, and SER-78 as well as the ligand. Due to the influence of the surrounding atoms of the proteins or the ligands, the key water molecules could be optimized to a reasonable site where they could form a more stable conformation by mediating the formation of the hydrogen bonds with the proteins or the ligands.

### 2.2. Validation of Multi-Body Docking Considering the Key Water Molecules as Fixed-Length Optimization Variables

#### 2.2.1. Docking Accuracy

**Figure 3:**
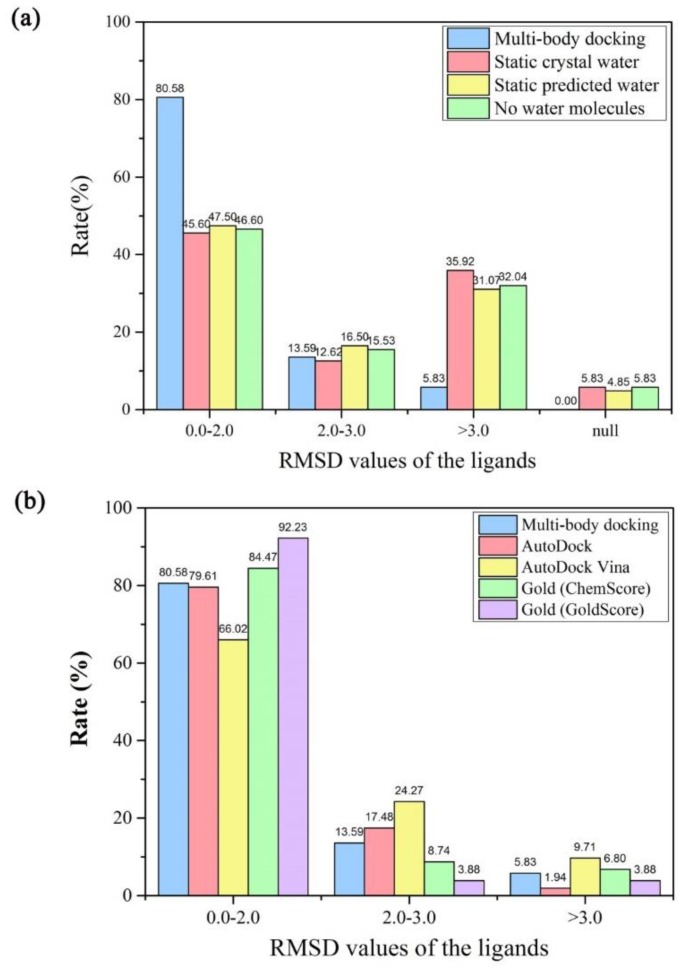
(**a**) Comparison of the best RMSD values for the recruit of the ligands under the four types of the docking simulations with different hydration strategies. (**b**) Comparison of the best RMSD values for the recruit of the ligands in the multi-body docking program considering the key water molecules as fixed-length optimization variables with the other docking programs.

Four types of the protein-ligand docking simulations were performed for each crystal structure to determine the effect of the water molecules on the docking accuracy. For the evaluation criterion of the best RMSD values for the recruit of the ligands smaller than 2.0 Å, the multi-body docking program yielded the highest success rate of 80.58% in the datasets compared with the other docking approaches (Figure 3a). These results suggested that the docking accuracy was improved significantly by considering the fixed numbers of the key water molecules in the multi-body docking simulations. Normally, when the water molecules were included as a static part of the protein in the docking simulations, they may occupy a fixed space of the binding site, which led to a steric hindrance for the translation or the rotation of the ligands. However, when there was no water molecule in the docking simulations, it may lead to a lack of the mediation of the water molecules between the protein and the ligand, and was also not beneficial to the conformation stability. While for the key water molecules that would move instantaneously during the docking simulations, they could not only move to a reasonable space in the binding sites but also form a more stable conformation by mediating the formation of the hydrogen bonds with the nearby proteins or the ligands. Therefore, the multi-body docking considering the key water molecules as fixed-length optimization variables could achieve a higher success rate of the best RMSD values for the recruit of the ligands.

**Figure 4:**
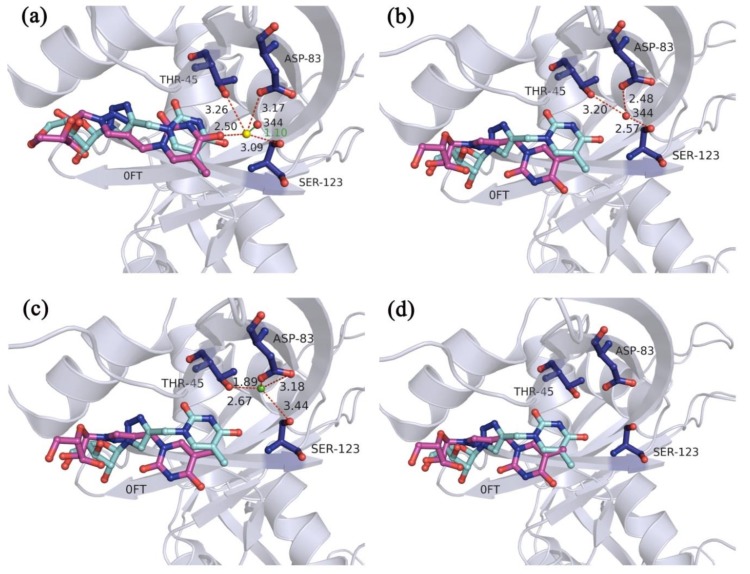
The docked poses of the best RMSD values for the recruit of the ligands in (**a**) the multi-body docking simulation considering the key water molecules as fixed-length optimization variables (**b**) the docking simulation when the experiment-determined water molecule W344 (red sphere) is included as a static part of the protein structure (**c**) the docking simulation when the predicted site of the water molecule (green sphere) is included as a static part of the protein structure and (**d**) the docking simulation without the water molecules of crystal structure 4G8Y. The ligands in the X-ray poses and the docked poses are shown as cyan and magenta ball-and-stick models, respectively. The hydrogen bonds between the water molecules and the amino acid residues or the ligands are represented by red dashed lines. The distance between the two water molecules is represented by a green solid line. Numbers beside the lines are the lengths.

Take the crystal structure 4G8Y (the crystal structure of ribonuclease A in complex with 5b) as an example to compare the performance of the four types of the docking simulations with different hydration strategies. In the multi-body docking simulation of crystal structure 4G8Y, the best RMSD value for the recruit of the ligand was 1.80 Å and the docked pose is shown in Figure 4a. In this docked pose, the optimized water molecule (yellow sphere) interacted with the oxygen atoms of the nearby three residues THR-45, ASP-83, and SER-123 as well as the ligand atom. And the distance between the optimized water molecule and the W344 was 1.10 Å. However, for the other three types of the docking simulations, the best RMSD values for the recruit of the ligands were 2.98 Å, 3.07 Å and 2.89 Å, respectively (Figure 4b–d). It could be seen that the rotatable bonds of the ligand had a large rotation due to the lack of the interactions between the key water molecule and the ligand. These results exhibited that the multi-body docking program considering the key water molecules as fixed-length optimization variables was a promising strategy to reproduce accurate docked poses of the ligands.

To further validate the docking accuracy of the multi-body docking program considering the key water molecules as fixed-length optimization variables, additional comparative tests were performed on AutoDock, AutoDock Vina, and Gold against the same datasets (Figure 3b). For the evaluation criterion of the best RMSD values for the recruit of the ligands with an RMSD value smaller than 2.0 Å, the multi-body docking program yielded a higher success rate than that of AutoDock (79.61%) and AutoDock Vina (66.02%), but slightly lower than that of Gold (ChemScore, 84.47%; GoldScore, 92.23%). This was due to a unique mechanism for placing the ligand in the binding site in Gold, which was based on fitting point [24]. Gold added fitting points to hydrogen bonding groups on protein and ligand, and mapped acceptor points on the ligand on donor points in the protein and vice versa. Additionally, GOLD generated hydrophobic fitting points in the protein cavity onto which ligand CH groups are mapped. Therefore, it generally could achieve better docking accuracy. Furthermore, as can be seen from the detailed comparison results of the four programs in Table 2, the multi-body docking program achieved an average RMSD value for the recruit of the ligands at 1.47 Å, whereas they were respectively 1.39 Å, 1.90 Å, 1.30 Å, and 0.86 Å in AutoDock, AutoDock Vina, Gold (ChemScore), and Gold (GoldScore). However, in terms of the docking accuracy, there were still a few docked poses of a higher RMSD value for the recruit of the ligand among the optimal solutions in the multi-body docking program. Take the crystal structures 1FCM and 1K97, whose best RMSD values for the recruit of the ligands were respectively 2.36 Å and 5.42 Å, for examples. The ligands both contained many polar atoms and may form more hydrogen bonds with the nearby polar atoms on the protein surface (Figure S1). However, the scoring function designed in the multi-body docking program had not yet taken into account the effect of the hydrogen bonding interactions, affecting its ability to the estimating of the binding affinity of the docked poses. Another possible reason may be that the ligands would situate at the entrance of a long and narrow protein pocket and thus may lead to a move to the outside.

#### 2.2.2. Cross-Docking Accuracy

The cross-docking accuracy was investigated on the presence and absence of the key water molecules by four protein targets: purine nucleoside phosphorylase (PNP), cyclooxygenase 1 (COX-1), HIV reverse transcriptase (HIVRT), and estrogen receptor antagonist (ER agonist). These proteins were selected from the DUD-E [25] as they had previously been determined if the judicious selection of a common set of water molecules would still result in improvements in the docking accuracy across a set of different ligands [7,26]. In addition, five representative structures for each protein target [26] were also adopted here and their PDB codes are listed in Table 3. All structures were aligned with respect to the template structure and the water molecules which were observed in all binding sites of the complexes would be selected in the multi-body docking simulations. As can be seen from the results of all docking simulations in Table 3, the inclusion of water molecules significantly improved the results of the receptors of 1B8N, 2AI1, 2AI2, 1C1B, 1RT1, 1VRT, and 1GWQ. In the case of the receptor of 1B8N (Figure S2a,b), the water molecule occupied a deep and wide space of the binding site, where it could interact with the polar atoms of the surrounding amino acids and the ligand. Therefore, the inclusion of the water molecules improved the recruit of the ligand. However, the docking simulations on the presence of the water molecules gave few worse results than those without the water molecules for the receptors of 3FUC and 2OYE. In the case of the receptor of 3FUC (Figure S2c,d), there was a long and narrow protein pocket which might lead to the space limitation for ligand optimization. At the same time, the water molecules may increase the space hindrance, leading to the poor optimization results. On the other hand, the presence of water molecules significantly improved the results of the ligands of 1B8N, 1B8O, 2AI1, 2AI2, 1Q4G, 1RT1, 1VRU, and 1L2I. And the poor docking results were obtained for the ligands of 3FUC, 1IGZ, 2AYL, and 2IOG in both docking simulations. Also, take 3FUC as an example, the ligand of 3FUC presented a long linear structure and contained many rotatable bonds, thus might have a steric clash with the surrounding protein side chains and the water molecules in the narrow binding site. On the whole, the success rates (the best RMSD value for the recruit of the ligand was within 2.0 Å) of 56.00%, 36.00%, 68.00%, and 40.00% were achieved in the cross-docking simulations on the presence of the water molecules of PNP, COX-1, HIVRT, and ER agonist, respectively, which were higher than the rates of 28.00%, 32.00%, 48.00%, and 36.00% in the docking simulation without the water molecules.

#### 2.2.3. Screening Efficiency

For the other evaluation criterion of the screening efficiency, all the docking programs were also assessed by those protein targets PNP, COX-1, HIVRT, and ER agonist. The detail results of the screening efficiency of the four programs are shown in Table 4. As can be seen from the true hits rates in the top 200 scorers, the multi-body docking program reproduced 42.00%, 64.00%, 32.56%, and 26.87% active inhibitors in the top 200 scorers of PNP, COX-1, HIVRT, and ER agonist, respectively, which were better than most of those in AutoDock, AutoDock Vina, and Gold. Furthermore, the enrichment factors (EFs) of the multi-body docking program were 3.62%, 5.70%, 11.54%, and 5.67% in the top 5% scorers of the ranked database of PNP, COX-1, HIVRT, and ER agonist, which also showed high performances compared with the other programs. Furthermore, the areas under the curve (AUC) for the receiver operating characteristic (ROC) plots were also adopted. The multi-body docking program yielded the highest AUC values of 0.68, 0.77, 0.76, and 0.82 for PNP, COX-1, HIVRT, and ER agonist, respectively, which indicated that the sensitivity of choosing active inhibitors over decoys of the multi-body docking program considering the key water molecules as fixed-length optimization variables was better than those of the other programs.

### 2.3. Validation of Multi-Body Docking Considering the Key Water Molecules as Variable-Length Optimization Variables

#### 2.3.1. Docking Accuracy

**Figure 5:**
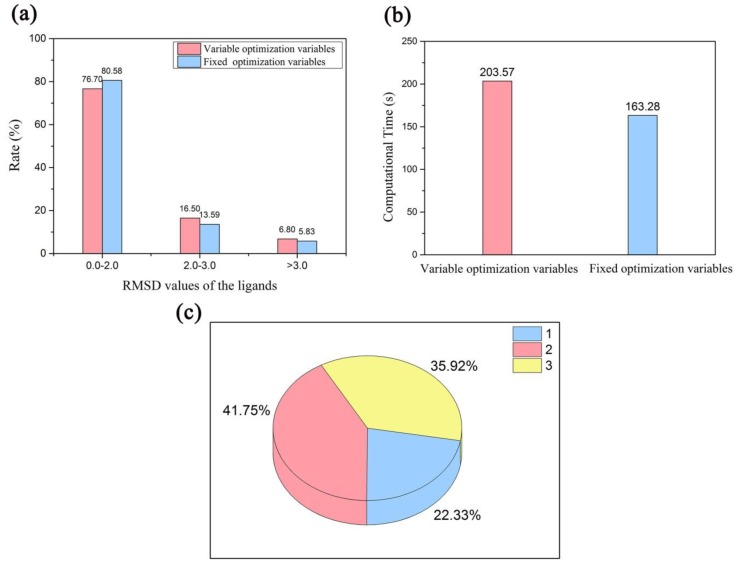
Comparison of the performance of the multi-body docking simulations considering the key water molecules as fixed-length optimization variables and variable-length optimization variables against the data sets in (**a**) the best RMSD values for the recruit of the ligands and (**b**) the computational time. (**c**) The proportions of the numbers of the optimized water molecules in the docked poses of the best RMSD values for the recruit of the ligands in the multi-body docking simulations considering the key water molecules as variable-length optimization variables.

A comparison between the multi-body docking simulations considering the key water molecules as fixed-length optimization variables and variable-length optimization variables was conducted (The detail results are shown in Table S3). The same parameters were set in both the docking simulations. From the Figure 5a and Table S2 we can see, the success rates of the best RMSD values for the recruit of the ligands with an RMSD value smaller than 2.0 Å and the average RMSD values for the recruit of the ligands in the two docking simulations were approximately equal. Additionally, the overall computational time of the multi-body docking simulations considering the key water molecules as variable-length optimization variables was about 1.25 times to that of the multi-body docking simulations considering the key water molecules as fixed-length optimization variables (Figure 5b). Furthermore, the proportions of one, two and three optimized water molecules in all docked poses of the best RMSD values for the recruit of the ligands in the multi-body docking simulations considering the key water molecules as variable-length optimization variables were 22.33%, 41.75%, and 35.92%, respectively (Figure 5c).

**Figure 6:**
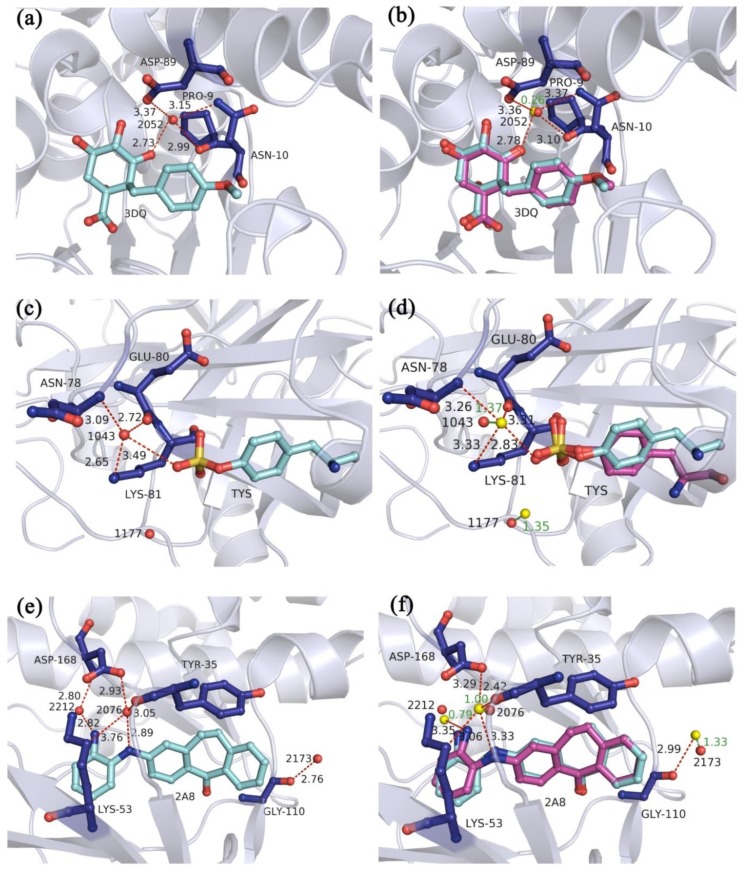
The hydrogen bond interactions of the experiment-determined water molecules in the binding sites of crystal structures (**a**) 4B6R, (**c**) 3SHC, and (**e**) 3ZYA. The docked poses of the best RMSD values for the recruit of the ligands in the multi-body docking program considering the key water molecules as variable-length optimization variables of crystal structure (**b**) 4B6R, (**d**) 3SHC, and (**f**) 3ZYA. The red and yellow spheres represent the experiment-determined water molecules and the optimized water molecules, respectively. The ligands in the X-ray poses and the docked poses are shown as cyan and magenta ball-and-stick models, respectively. The hydrogen bonds between the water molecules and the amino acid residues or the ligands are represented by red dashed lines. The distances between the two water molecules are represented by green solid lines. Numbers beside the lines are the lengths.

Take the crystal structures 4B6R, 3SHC, and 3ZYA, of which the numbers of the optimized water molecules in the docked poses of the best RMSD values for the recruit of the ligands were respectively one, two, and three, for examples. For the crystal structure 4B6R (Figure 6a), of which only one optimized water molecule was in the optimal docked pose, the experiment-determined water molecule W2052 (red sphere) simultaneously interacted with the residues PRO-9, ASN-10, and ASP-89 as well as the ligand atom. After the optimization, the best RMSD value for the recruit of the ligand was 0.80 Å and the pose of the docked ligand highly coincided with the ligand in the crystal structure (Figure 6b). Meanwhile, the optimized water molecule (yellow sphere) was 0.26 Å away from the W2052. And it interacted with the same three residues PRO-9, ASN-10, and ASP-89 and the ligand atom.

For the crystal structure 3SHC, there were two optimized water molecules in the optimal docked pose. And in this optimal docked pose, the best RMSD value for the recruit of the ligand was 1.69 Å and the rotatable bonds at one end of the ligand had a slight rotation (Figure 6d). In addition, one of the optimized water molecules (yellow spheres) was 1.37 Å away from the experiment-determined water molecule W1043, and it interacted with the three residues ASN-78, GLU-80, and LYS-81 and the ligand atom (Figure 6c). Another optimized water molecule was 1.35 Å away from the experiment-determined water molecule W1177.

Similarly, for the crystal structure 3ZYA, there were three optimized water molecules in the optimal docked pose. In this optimal docked pose, the best RMSD value for the recruit of the ligand was 0.50 Å (Figure 6f) and the pose of the docked ligand highly coincided with the ligand in the crystal structure. Furthermore, the minimum distances between the optimized water molecules and the experiment-determined water molecules W2076, W2173 and W2212 were 1.00 Å, 1.33 Å, and 0.79 Å, respectively. Among them, one of the optimized water molecules interacted with the residues TYR-35, LYS-53, and ASP-168 as well as the ligand atom in the binding site, either was the W2076. The other optimized water molecules had a smaller translational motion from the W2173 and W2212.

Given the above, these results suggested that the multi-body docking with the variable numbers of the water molecules was capable of reproducing accurate docked poses of the ligands. Meanwhile, the sites of the optimized water molecules were the potential hydration sites in the binding sites of crystal structures.

#### 2.3.2. Cross-Docking Accuracy

The cross-docking accuracy of the multi-body docking considering the key water molecules as variable-length optimization variables was also investigated on the presence and absence of the key water molecules by the same four protein targets PNP, COX-1, HIVRT, and ER agonist. As can be seen from the results of the docking simulations in Table 5, the docking simulations on the presence of the water molecules significantly improved the results of the receptors of 1B8N, 2AI1, 2AI2, 1C1B, 1RT1, 1VRT, 1GWQ, and 2IOG, and the results of the ligands of 1B8N, 1B8O, 2AI1, 2AI2, 1Q4G, 1C1B, 1RT1, 1VRU, 1L2I, and 1XPC. On the whole, the success rates (the best RMSD value for the recruit of the ligand was within 2.0 Å) of 60.00%, 36.00%, 60.00%, and 44.00% were achieved in the cross-docking simulations on the presence of the water molecules of PNP, COX-1, HIVRT, and ER agonist, respectively, which were higher than the rates of 28.00%, 32.00%, 48.00%, and 36.00% in the docking simulations without the water molecules. And the success rates of the multi-body docking considering the key water molecules as variable-length optimization variables for the four protein targets were comparable to those of the multi-body docking considering the key water molecules as fixed-length optimization variables (Table 3).

#### 2.3.3. Screening Efficiency

The detailed comparison of the screening efficiency of the multi-body docking considering the key water molecules as fixed-length optimization variables and variable-length optimization variables are shown in Table 6. As can be seen from the scoring positions of active inhibitors among all the compounds, the multi-body docking considering the key water molecules as variable-length optimization variables reproduced 56.00%, 56.00%, 48.84%, and 25.37% in the top 200 scorers of PNP, COX-1, HIVRT and ER agonist, which showed a relatively high accuracy to those of the multi-body docking considering the key water molecules as fixed-length optimization variables. Furthermore, the EFs of the multi-body docking considering the key water molecules as variable-length optimization variables were 7.25%, 8.14%, 5.12%, and 5.07% in the top 5% scorers of the ranked database of PNP, COX-1, HIVRT and ER agonist, which was also approximately equal to those of the multi-body docking considering the key water molecules as fixed-length optimization variables. In addition, both the multi-body docking simulations yielded higher AUC values for the four protein targets. Above all, those docking simulations considering the key water molecules as fixed-length optimization variables or variable-length optimization variables demonstrated high screening efficiencies.

## 3. Methods

### 3.1. Model of the Multi-Body Interaction

**Figure 7:**
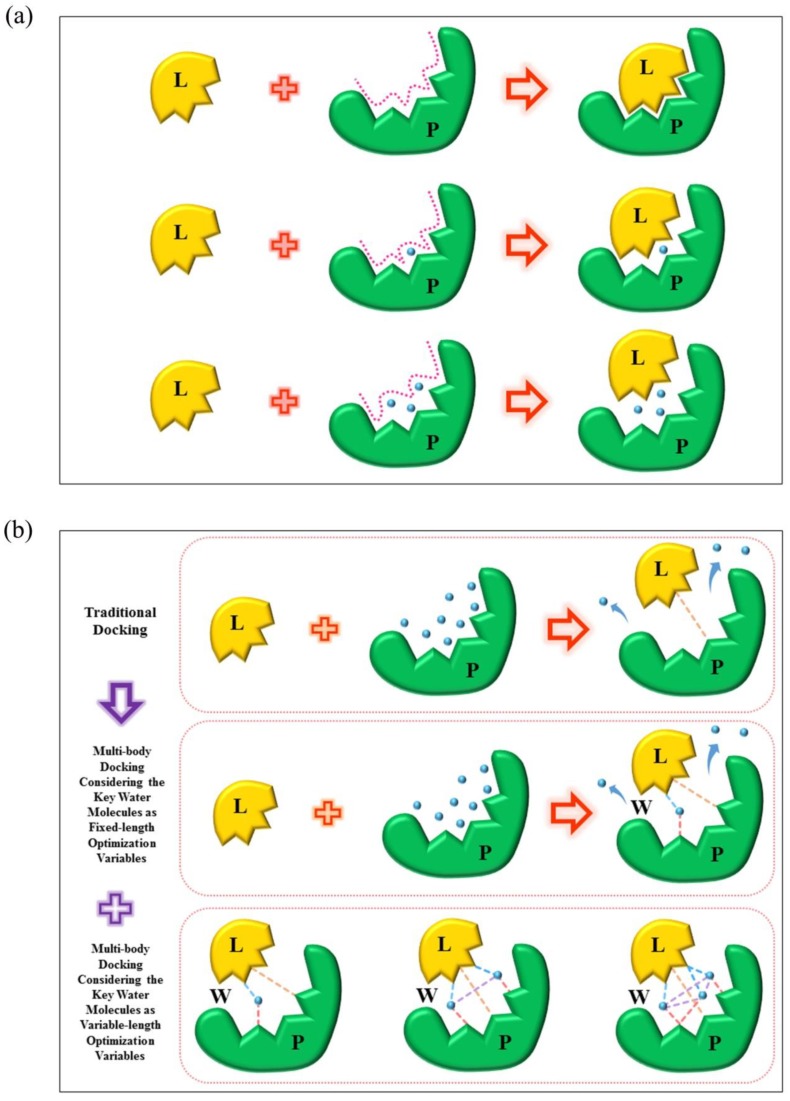
(**a**) Effect of the key water molecules on the shape of the binding site. (**b**) The multi-body interaction model, where L, P, and W represent the ligand, protein and water molecule(s); and the orange, pink, blue and purple dashed lines represent the protein-ligand, protein-water, ligand-water, and water-water interactions, respectively.

Water molecules found in crystal structures contribute to the flexibility of the shape of the binding sites. As can be seen in Figure 7a, in traditional molecular docking, the ligand would be closely docked to the protein active sites by shape matching and the electrostatic complementary interactions without the effect of the water molecules. However, when the key water molecules were included in the docking simulations and would move instantaneously, it may lead to a reduction of the solvent accessible surface area by occupying a certain space of the binding site. Moreover, with the increasing number of the key water molecules, the shape of the binding site might change completely. Therefore, how to explicitly consider the role of the key water molecules in docking had become a difficult issue.

To solve the above-mentioned problem, we have designed the multi-body interactions model considering the numbers of the key water molecules as the optimization variables in docking simulations (Figure 7b). Different from the traditional molecular docking without the water molecules, this multi-body interactions model took into account the effect of the key water molecules in two separate ways: (i) the numbers of the key water molecules were included as fixed-length optimization variables, that is, the fixed numbers of the key water molecules would participate in the docking simulations; (ii) the numbers of the key water molecules were included as variable-length optimization variables, that is, the variable numbers of the key water molecules would participate in the docking simulations. Through the above measures, the multi-body interaction model of protein-water-ligand was designed.

### 3.2. Multi-Body Interaction Considering the Key Water Molecules as Variable-Length Optimization Variables

With the special consideration of the numbers of the key water molecules as variable-length optimization variables in the multi-body docking simulations, a genetic algorithm with the unequal chromosome cross-over was employed. In this genetic algorithm, every individual in evolution was coded as a chromosome by real-number coding; each chromosome was composed of multiple genes, and one gene on a chromosome represented a degree of freedom. The degrees of freedom on each individual chromosome included the state variables of the translation and rotation of the entire ligand, the torsion angles of *n* rotatable bonds of the ligand, and the state variables of the translation of *m* water molecules for the orientation search. Besides that, an extra degree of freedom was defined as the sign for identifying the variable numbers of the water molecules in the multi-body docking simulations was also added. 

**Figure 8:**
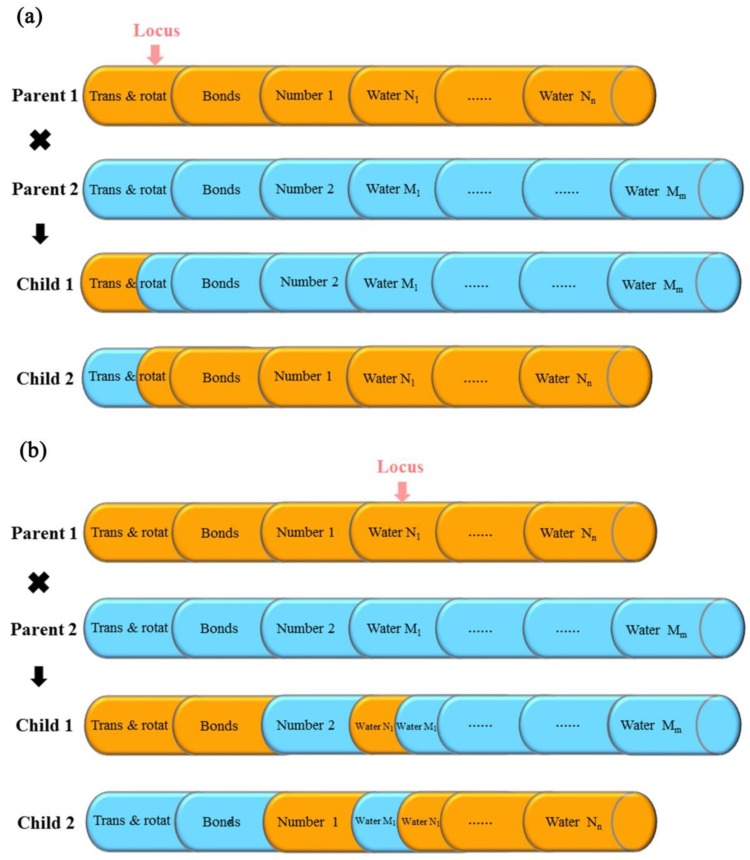
The mechanism of the unequal chromosome cross-over. (**a**) The operation of the unequal chromosome cross-over when the random gene locus sited on the degrees of freedom of the ligand. (**b**) The operation of the unequal chromosome cross-over when the random gene locus sited on the degrees of freedom of the water molecules. Trans and rotat stand for the state variables of the translation and rotation of the entire ligand, respectively; bonds stand for the state variables of the torsion angles of the rotatable bonds of the ligand; Number 1 and Number 2 represent the signs of the numbers of the water molecules; Water N_n_ (Water M_m_) stands for the state variables of the translation of the number N_n_ (M_m_) of the water molecule. Locus represents the random gene locus for the cross-over operation.

Based on the compositions of the degrees of freedom, the detailed mechanism of the unequal chromosome cross-over was as followed (Figure 8). (i) For any two parent chromosomes in the cross-over operation, the numbers of the degrees of freedom were compared; (ii) The parent chromosome with a smaller number of the degrees of freedom was selected (if the numbers of the degrees of freedom for the two parent chromosomes were equal, one of the parent chromosomes would be chosen randomly); (iii) The gene locus for the cross-over operation was randomly chosen from the loci of the parent chromosome with a smaller number of the degrees of freedom; (iv) The cross-over operation would be performed in two independent ways: when the random gene locus for the cross-over operation sited on the degrees of freedom of the ligand (the state variables of the translation and rotation of the ligand, or the state variables of the torsion angles of the rotatable bonds of the ligand for the conformational search), the two parent chromosomes would exchange all the degrees of freedom after the random gene locus directly (Figure 8a). On the other hand, when the random gene locus for the cross-over operation sited on the degrees of freedom of the water molecules (the sign of the number of the water molecules or the state variables of the translation of the water molecules), the two parent chromosomes would exchange the degrees of freedom of the signs as well as the degrees of freedom after the random gene locus (Figure 8b).

### 3.3. Design of Multi-Body Docking 

#### 3.3.1. Multi-Objective Optimization Model and Algorithm for Multi-Body Docking 

To properly consider the water molecules that mediated between the ligands and the proteins, we designed a multi-objective molecular docking optimization model based on a multi-objective optimization algorithm, non-dominated sorting genetic algorithm II (NSGA II) [27]. The multi-objective molecular docking optimization model contained two objective functions $f_{1}\left(x\right)$ and $f_{2}\left(x\right)$ which were derived from the force-field-based scoring functions, and a set of decision variables (x) subjected to the conformational space of **S** as follows:

(1) $$\begin{array}{cc} \min & y=f\left(x\right)=\left(f_{1}\left(x\right),f_{2}\left(x\right)\right)\\ \mathrm{subject} & \mathrm{to}\;e\left(x\right)=\left(e_{1}\left(x\right),e_{2}\left(x\right),\ldots ,e_{k}\left(x\right)\right)\leq 0\\ \mathrm{where} & x={\left\{x_{1},x_{2},\ldots ,x_{m}\right\}}^{T}\in S \end{array}$$

where x = {*x*_1_, *x*_2_, …, *x*_m_} ^T^ = {*T*_x_, *T*_y_, *T*_z_, *R*_x_, *R*_y_, *R*_z_, *R*_b1_, *…*, *R*_bn_, *W*_x1_, *W*_y1_, *W*_z1_, *…*, *W*_xm_, *W*_ym_, *W*_zm_} ^T^, in which (*T*_x_, *T*_y_, *T*_z_) and (*R*_x_, *R*_y_, *R*_z_) are the state variables of the translation and rotation, respectively, of the entire ligand for the orientation search; (*R*_b1_, *…*, *R*_bn_) are the torsion angles of the *n* rotatable bonds of the ligand for the conformational search; and (*W*_x1_, *W*_y1_, *W*_z1_, *…*, *W*_xm_, *W*_ym_, *W*_zm_) are the state variables of the translation of the *m* water molecules for the orientation search. The constraints of these decision variables (*e*_1_(x), *e*_2_(x), …, *e*_k_(x)) are as follows:

(2) $$X_{low}\leq T_{x},W_{x1},\ldots ,W_{xm}\leq X_{up}\;Y_{low}\leq T_{y},W_{y1},\ldots ,W_{ym}\leq Y_{up}\;Z_{low}\leq T_{z},W_{z1},\ldots ,W_{zm}\leq Z_{up}\;0\leq R_{x},R_{y},R_{z},R_{b1},\ldots ,R_{bn}\leq 2\pi$$

where *X_up_*(*X_low_*), *Y_up_*(*Y_low_*) and *Z_up_*(*Z_low_*) are the upper (lower) bounds of the translational motion of the ligand or the water molecules. 

#### 3.3.2. Scoring Function Designed for Multi-Body Docking. 

The force-field-based scoring function is designed as follows:

(3) $$E=E_{pro-lig}+E_{pro-wat}+E_{lig-wat}+E_{wat-wat}+E_{lig}$$

where *E_pro−lig_*, *E_pro−wat_*, *E_lig−wat_*, and *E_wat__−wat_* are the protein-ligand, protein-water, ligand-water, and water-water interaction energy terms, respectively, and *E_lig_* is the intramolecular ligand conformational energy term. And when the number of the water molecules that take part in molecular docking is only one, the term *E_wat__-wat_* would be ignored. The energy terms in Equation (3) are further utilized to design as the objective functions $f_{1}\left(x\right)$ and $f_{2}\left(x\right)$:

(4) $$f_{1}\left(x\right)=E_{pro-lig}+E_{pro-wat}$$

(5) $$f_{2}\left(x\right)=E_{lig-wat}+E_{wat-wat}+E_{lig}$$

where

(6) $$E_{pro-lig}=\overset{pro}{\underset{i}{\sum }}\overset{lig}{\underset{j}{\sum }}E_{i,j}^{\mathrm{vdW}}\left(r\right)+\overset{pro}{\underset{i}{\sum }}\overset{lig}{\underset{j}{\sum }}E_{i,j}^{\mathrm{es}}\left(r\right)$$

(7) $$E_{pro-wat}=\overset{pro}{\underset{i}{\sum }}\overset{wat}{\underset{j}{\sum }}E_{i,j}^{\mathrm{vdW}}\left(r\right)+\overset{pro}{\underset{i}{\sum }}\overset{wat}{\underset{j}{\sum }}E_{i,j}^{\mathrm{es}}\left(r\right)$$

(8) $$E_{lig-wat}=\overset{lig}{\underset{i}{\sum }}\overset{wat}{\underset{j}{\sum }}E_{i,j}^{\mathrm{vdW}}\left(r\right)+\overset{lig}{\underset{i}{\sum }}\overset{wat}{\underset{j}{\sum }}E_{i,j}^{\mathrm{es}}\left(r\right)$$

(9) $$E_{wat-wat}=\overset{wat}{\underset{i}{\sum }}\overset{wat}{\underset{j\neq i}{\sum }}E_{i,j}^{\mathrm{vdW}}\left(r\right)+\overset{wat}{\underset{i}{\sum }}\overset{wat}{\underset{j\neq i}{\sum }}E_{i,j}^{\mathrm{es}}\left(r\right)$$

(10) $$E_{lig}=\overset{lig}{\underset{i}{\sum }}\overset{lig}{\underset{j\neq i}{\sum }}E_{i,j}^{\mathrm{vdW}}\left(r\right)+\overset{lig}{\underset{i}{\sum }}\overset{lig}{\underset{j\neq i}{\sum }}E_{i,j}^{\mathrm{es}}\left(r\right)$$

where $E_{i,j}^{\mathrm{vdW}}\left(r\right)$ and $E_{i,j}^{\mathrm{es}}\left(r\right)$ in Equations (6)–(9) are the van der Waals (vdW) and electrostatic interaction energies between atom *i* and atom *j* at a distance *r*, respectively; $E_{i,j}^{\mathrm{vdW}}\left(r\right)$ and $E_{i,j}^{\mathrm{es}}\left(r\right)$ in Equation (10) represent the internal vdW and electrostatic interaction energies of the nonbonded atom pair *i*, *j* of the ligand at a distance r. The vdW energy and electrostatic energy are respectively calculated using the Lennard-Jones 6–12 potential and the coulombic potential. 

### 3.4. Properties of Multi-Body Docking

**Figure 9:**
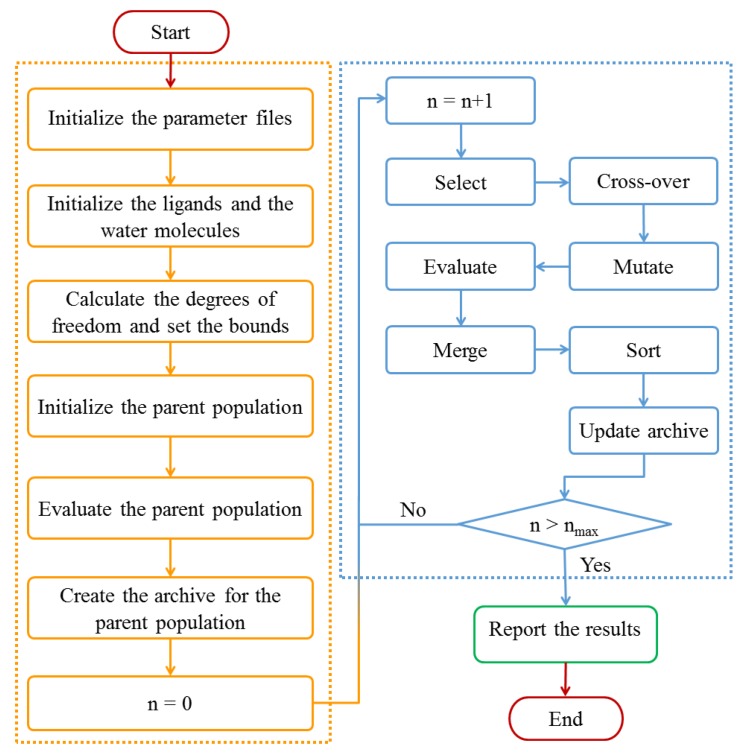
Flowchart of the multi-body docking program.

To obtain the optimal binding poses of the multi-body docking simulations, we designed a workflow that consists of several calculation and optimization steps, as shown in Figure 9. The multi-body docking approach was coded in C++, and the following parameters were adopted: the active sites included protein residues within a sphere with a radius of 20.0 Å centered on the center of the ligands in the experimental crystal structures [28]. For each NSGA II optimization run, 500 generations were performed on an initial population with a size of 2000, and the operator weights for cross-over and mutation were set to 0.9 and 0.1, respectively. Detailed results for the parameters comparison in NSGA II are shown in Appendix A (Supplementary Materials). Furthermore, the ranges of the translation and rotation of the ligands were set to ±4.0 Å and ±3.14 rad, respectively. And the range of the translation for the water molecules was ±2.0 Å.

### 3.5. Preparation of the Data Sets 

The test sets from the methods of the tetrahedron-water-cluster model [3] and the AutoDock hydrated docking [29] formed the available dataset in this multi-body docking simulations. The following screening criteria were added to narrow down the datasets: (i) X-ray crystal structures with a resolution smaller than 2.0 Å; (ii) no alternate or distorted configurations; (iii) exclude the ligands with more than 12 rotatable bonds to limit the search space complexity [29]. The final resulting set consisted of 103 complexes (see Table S1 for a comprehensive overview of the complexes included).

*Preparation of protein structures.* For the protein structures, all hydrogen atoms were added using Sybyl (Tripos Inc., Princeton, NJ, USA). The proteins were protonated and assigned Amber ff99SB force field parameters [30].

*Preparation of ligands.* The ligands were extracted from the complexes, and all bonds and atom types were checked for consistency. All hydrogen atoms and Gasteiger-Marsili atomic partial charges [31] were added, respectively.

*Preparation of water molecules.* For the water molecules, the predicted hydration sites by the tetrahedron-water-cluster model [3] were retained as the original sites in the multi-body docking simulations. And about 28.16% of the predicted hydration sites were within 1.0 Å away from the sites of the water molecules in the crystal structures (see Table S1 for a comprehensive overview of the distances between them). All hydrogen atoms and Gasteiger-Marsili atomic partial charges were also added.

### 3.6. Validation of Multi-Body Docking 

In order to determine the effect of the key water molecules on the accuracy performance of the docking simulations, four types of the docking programs with different hydration strategies were performed for each crystal structure. The main features of these simulations are as followed: (i) Multi-body docking: the numbers of the key water molecules were treated as fixed-length optimization variables in the multi-body docking simulations; (ii) Static crystal water: the water molecules in crystal structures were included as a static part of the protein structures in the docking simulations; (iii) Static predicted water: the water molecules for the sites predicted by the tetrahedron-water-cluster model were included as a static part of the protein structures in the docking simulations; (iv) No water molecules: docking simulations without the water molecules.

Furthermore, the performance of the multi-body docking program was also compared with the other popular docking programs: AutoDock [32,33] (version 4.2), AutoDock Vina [34] and Gold (version 4.1.2) [10].

AutoDock used the Lamarckian genetic algorithm as the conformational search algorithm and a force field and hydration docking method that enabled the automated prediction of the water molecules mediating the binding of ligands with target proteins [32]. The binding pocket, defined as a three-dimensional grid with dimensions of 70 × 70 × 70 points along the x, y, and z axes, was centered on the ligand in the experimental complex with a grid spacing of 0.375 Å [35]. For the other options, the default values were retained, and the top 10 ranked binding poses for each ligand were reserved.

Additionally, AutoDock Vina, a reported program which improved the accuracy of the binding mode predictions and achieved approximately two orders of magnitude speed-up compared with AutoDock 4, was also adopted. AutoDock Vina used a sophisticated gradient optimization method in its local optimization procedure. And the key water molecules were kept as part of the ligands during the docking simulations. In addition, a docking grid with a default size of 22.5 × 22.5 × 22.5 Å^3^ and the top 30 ranked binding poses for each ligand were reserved.

GOLD, another famous docking program, used a genetic algorithm to explore water mediations and displacements in the docking simulations. The key water molecules were picked out and set to rotate around its three principal axes freely during the docking simulations. Meanwhile, a constant penalty, σ_p_, representing the loss of rigid-body entropy, was added for the water molecules that are switched on, hence rewarding the water displacement. In each genetic algorithm run, a default population size of 100 and a number of 100,000 generations were used for 30 independent searching and optimization runs. And the two scoring functions ChemScore and GoldScore [10] were respectively chosen for this study. 

Besides, a comparison between the multi-body docking simulations considering the numbers of the key water molecules as the fixed-length optimization variables and the variable-length optimization variables was also conducted to validate its performance.

## 4. Conclusions

A multi-body docking program that incorporated the fixed or the variable number of the key water molecules in docking simulations was designed in this study. This program employed a multi-objective optimization algorithm to identify the binding poses of protein-water-ligand. And a force-field-based hydration-specific scoring function was designed to evaluate their binding poses. Moreover, the performance of the multi-body docking program was evaluated in aspects of the docking accuracy, cross-docking accuracy, and screening efficiency. When the numbers of the key water molecules were treated as the fixed-length optimization variables, the multi-body docking program achieved a success rate of 80.58% for the evaluation criterion of the best RMSD values for the recruit of the ligands smaller than 2.0 Å. The success rates of 56.00%, 36.00%, 68.00%, and 40.00% were achieved in the cross-docking simulations on the presence of the water molecules of PNP, COX-1, HIVRT, and ER agonist. The highest AUC values of 0.68, 0.77, 0.76, and 0.82 for PNP, COX-1, HIVRT, and ER agonist were obtained in the screening efficiency. All of the results revealed that the multi-body docking considering the key water molecules as fixed-length optimization variables was performed well compared with the other programs. On the other side, when the numbers of the key water molecules were treated as the variable-length optimization variables in the multi-body docking simulations, the program obtained comparative performance under the same three evaluation criterions. In the following work, we will continue to optimize the scoring function to improve the performance of the multi-body docking program. 

## Figures and Tables

**Table 1:** The best RMSD values for the recruit of the ligands and the computational time in the multi-body docking simulations considering the key water molecules as fixed-length optimization variables.

N_rot_^a^	N_com_^b^	Min RMSD (Å)	Max RMSD (Å)	Avg RMSD (Å)	Min Time (s)	max Time (s)	Avg Time (s)
1–5	49	0.14	5.42	1.51	47.70	275.06	129.01
6–10	42	0.16	3.46	1.28	44.70	451.77	181.18
11–15	12	0.29	5.46	1.77	86.54	464.05	240.54
all	103	0.14	5.46	1.47	44.70	464.05	166.19

^a^ Number of rotatable bonds of the ligands; ^b^ Number of the complexes.

**Table 2:** Comparisons of the best RMSD values for the recruit of the ligands over the four docking programs.

		N_rot_^a^ (N_com_^b^)	1–5			6–10			11–15			All Ligands		
	RMSD (Å)		(49 Cases)			(42 Cases)			(12 Cases)			(103 Cases)		
Method			min	max	avg	min	max	avg	min	max	avg	min	max	avg
Multi-body docking			0.14	5.42	1.51	0.16	3.46	1.28	0.29	5.46	1.77	0.14	5.46	1.47
AutoDock			0.14	3.68	1.27	0.39	6.93	1.46	0.68	2.96	1.65	0.14	6.93	1.39
AutoDock Vina			0.22	7.03	1.88	0.48	5.84	1.86	1.19	3.27	2.16	0.22	7.03	1.90
Gold (ChemScore)			0.14	11.36	1.31	0.17	4.99	1.14	0.20	9.14	1.80	0.14	11.36	1.30
Gold (GoldScore)			0.22	4.75	0.89	0.35	3.41	0.96	0.35	4.68	1.20	0.22	4.75	0.86

^a^ Number of rotatable bonds of the ligands; ^b^ Number of the complexes.

**Table 3:** Results of the cross-docking simulations considering the key water molecules as fixed-length optimization variables and without the water molecules.

**Protein Target**			L ^a^	**1B8N**	**1B8O**	**2AI1**	**2AI2**	**3FUC**
		V ^b^						
	R ^c^							
PNP	1B8N			0.19 (5.71)	0.30 (0.62)	1.72 (6.40)	1.26 (6.46)	4.88 (4.69)
	1B8O			0.28 (0.35)	0.16 (0.30)	1.17 (1.25)	1.10 (1.51)	4.83 (4.55)
	2AI1			1.09 (0.98)	0.65 (5.12)	0.56 (6.21)	0.43 (6.22)	5.46 (6.36)
	2AI2			2.62 (5.70)	0.88 (5.78)	3.61 (7.01)	0.55 (0.45)	5.00 (7.80)
	3FUC			5.14 (5.07)	5.17 (5.12)	3.48 (7.66)	4.62 (3.88)	4.62 (7.29)
COX-1				**1EQG**	**1IGZ**	**1Q4G**	**2AYL**	**2OYE**
	1EQG			0.93 (0.40)	3.61 (5.01)	0.25 (0.30)	2.19 (2.88)	2.87 (4.13)
	1IGZ			3.15 (3.77)	3.48 (4.70)	3.01 (3.10)	3.39 (3.85)	3.71 (3.84)
	1Q4G			0.43 (0.48)	5.41 (4.70)	0.20 (0.48)	2.45 (5.22)	3.81 (2.78)
	2AYL			1.00 (0.54)	4.89 (5.08)	0.24 (0.26)	2.18 (4.92)	2.67 (3.23)
	2OYE			2.00 (1.91)	4.84 (5.33)	1.83 (2.29)	5.66 (2.28)	1.33 (1.32)
HIVRT				**1C1B**	**1RT1**	**1RTH**	**1VRT**	**1VRU**
	1C1B			2.34 (5.04)	1.79 (4.69)	1.94 (1.95)	1.37 (1.38)	1.47 (1.92)
	1RT1			0.99 (2.00)	0.90 (3.37)	1.81 (1.72)	1.42 (1.71)	1.99 (5.63)
	1RTH			3.14 (4.93)	2.61 (3.42)	0.32 (0.36)	0.38 (0.38)	2.29 (5.11)
	1VRT			2.98 (3.79)	1.86 (3.79)	1.59 (1.66)	0.30 (0.31)	2.11 (4.70)
	1VRU			2.42 (3.67)	3.81 (3.87)	1.20 (1.19)	0.84 (0.91)	2.00 (2.07)
ER agonist				**1GWQ**	**1GWR**	**1L2I**	**1XPC**	**2IOG**
	1GWQ			0.58 (0.78)	0.51 (0.86)	0.59 (6.70)	3.90 (2.30)	4.52 (5.18)
	1GWR			0.72 (0.75)	0.18 (0.30)	0.51 (0.59)	3.29 (5.75)	3.18 (2.77)
	1L2I			0.44 (0.59)	0.52 (0.59)	0.55 (0.45)	3.34 (6.01)	4.98 (4.83)
	1XPC			3.24 (5.41)	2.63 (4.99)	4.29 (5.08)	1.88 (1.76)	3.74 (4.80)
	2IOG			2.69(2.64)	3.53 (4.07)	4.11 (4.39)	2.26 (2.56)	3.32 (5.67)

^a^ ligands; ^b^ the best RMSD values for the recruit of the ligands in the cross-docking simulations on the presence of the water molecules (the best RMSD values for the recruit of the ligands in the cross-docking simulations without the water molecules). RMSD values in Å.; ^c^ Receptors.

**Table 4:** Comparison of the performance of the four docking programs in virtual screening.

Protein Targets	Programs	True Hits Rate in the Top 200 Scorers	EF in the Top 5% Scorers	AUC Values
PNP	Multi-body docking	42.00%	3.62%	0.68
	AutoDock	26.00%	3.62%	0.61
	AutoDock Vina	16.00%	0.00%	0.44
	Gold (ChemScore)	10.00%	0.80%	0.33
	Gold (GoldScore)	14.00%	2.41%	0.38
COX-1	Multi-body docking	64.00%	5.70%	0.77
	AutoDock	4.00%	0.81%	0.21
	AutoDock Vina	64.00%	9.77%	0.68
	Gold (ChemScore)	32.00%	4.88%	0.53
	Gold (GoldScore)	12.00%	0.00%	0.45
HIVRT	Multi-body docking	32.56%	11.54%	0.76
	AutoDock	27.90%	6.41%	0.50
	AutoDock Vina	9.30%	1.28%	0.26
	Gold (ChemScore)	2.33%	0.00%	0.32
	Gold (GoldScore)	39.53%	14.10%	0.59
ER agonist	Multi-body docking	26.87%	5.67%	0.82
	AutoDock	29.85%	4.17%	0.75
	AutoDock Vina	50.75%	8.35%	0.80
	Gold (ChemScore)	0.00%	0.00%	0.11
	Gold (GoldScore)	1.49%	0.00%	0.28

**Table 5:** Results of the cross-docking simulations considering the key water molecules as variable-length optimization variables and without the water molecules.

**Protein Target**			**L ^a^**	**1B8N**	**1B8O**	**2AI1**	**2AI2**	**3FUC**
		**V ^b^**						
	**R ^c^**							
PNP	1B8N			0.44 (5.71)	0.72 (0.62)	1.17 (6.40)	1.21 (6.46)	4.15 (4.69)
	1B8O			0.25 (0.35)	0.29 (0.30)	1.09 (1.25)	0.96 (1.51)	4.77 (4.55)
	2AI1			0.73 (0.98)	0.62 (5.12)	0.38 (6.21)	0.54 (6.22)	6.86 (6.36)
	2AI2			0.82 (5.70)	0.71 (5.78)	6.85 (7.01)	0.75 (0.45)	6.83 (7.80)
	3FUC			2.96 (5.07)	2.98 (5.12)	4.32 (7.66)	4.71 (3.88)	5.68 (7.29)
COX-1				**1EQG**	**1IGZ**	**1Q4G**	**2AYL**	**2OYE**
	1EQG			0.22 (0.40)	4.57 (5.01)	0.21 (0.30)	2.34 (2.88)	4.60 (4.13)
	1IGZ			3.53 (3.77)	4.84 (4.70)	2.89 (3.10)	3.74 (3.85)	4.46 (3.84)
	1Q4G			0.44 (0.48)	4.78 (4.70)	0.44 (0.48)	2.42 (5.22)	2.80 (2.78)
	2AYL			1.33 (0.54)	5.66 (5.08)	0.85 (0.26)	2.26 (4.92)	3.92 (3.23)
	2OYE			2.00 (1.91)	4.24 (5.33)	1.96 (2.29)	3.23 (2.28)	1.49 (1.32)
HIVRT				**1C1B**	**1RT1**	**1RTH**	**1VRT**	**1VRU**
	1C1B			1.54 (5.04)	2.68 (4.69)	1.83 (1.95)	1.24 (1.38)	1.77 (1.92)
	1RT1			1.06 (2.00)	1.31 (3.37)	1.82 (1.72)	0.97 (1.71)	2.56 (5.63)
	1RTH			3.56 (4.93)	3.11 (3.42)	0.36 (0.36)	0.40 (0.38)	2.79 (5.11)
	1VRT			2.73 (3.79)	1.92 (3.79)	1.73 (1.66)	0.30 (0.31)	2.46 (4.70)
	1VRU			2.83 (3.67)	3.10 (3.87)	1.20 (1.19)	0.78 (0.91)	2.00 (2.07)
ER agonist				**1GWQ**	**1GWR**	**1L2I**	**1XPC**	**2IOG**
	1GWQ			0.38 (0.78)	0.60 (0.86)	0.62 (6.70)	3.28 (2.30)	5.67 (5.18)
	1GWR			0.50 (0.75)	0.14 (0.30)	0.57 (0.59)	3.77 (5.75)	4.48 (2.77)
	1L2I			0.32 (0.59)	0.56 (0.59)	0.47 (0.45)	3.87 (6.01)	4.34 (4.83)
	1XPC			5.28 (5.41)	5.15 (4.99)	4.40 (5.08)	1.20 (1.76)	3.89 (4.80)
	2IOG			2.63 (2.64)	2.54 (4.07)	4.02 (4.39)	1.76 (2.56)	3.99 (5.67)

^a^ ligands; ^b^ the best RMSD values for the recruit of the ligands in the cross-docking simulations on the presence of the water molecules (the best RMSD values for the recruit of the ligands in the cross-docking simulations without the water molecules). RMSD values in Å; ^c^ Receptors.

**Table 6:** Comparison of the performance of the multi-body docking considering the key water molecules as fixed-length optimization variables and variable-length optimization variables in virtual screening.

Protein Targets	Fixed/Variable ^a^	True Hits Rate in the Top 200 Scorers	EF in the Top 5% Scorers	AUC Values
PNP	Fixed	42.00%	3.62%	0.68
	Variable	56.00%	7.25%	0.79
COX-1	Fixed	64.00%	5.70%	0.77
	Variable	56.00%	8.14%	0.68
HIVRT	Fixed	32.56%	11.54%	0.76
	Variable	48.84%	5.12%	0.75
ER agonist	Fixed	26.87%	5.67%	0.82
	Variable	25.37%	5.07%	0.56

^a^ Fixed stands for the multi-body docking considering the key water molecules as fixed-length optimization variables; Variable stands for the multi-body docking considering the key water molecules as variable-length optimization variables.

## Supplementary Materials

The following are available online. Figure S1: The docked poses of the best RMSD values for the recruit of the ligands in the multi-body docking program considering the key water molecules as fixed-length optimization variables of crystal structure (a) 1FCM and (b) 1K97, Figure S2: The poses of crystal structures 1B8N (a,b) and 3FUC (c,d), Appendix A: Comparison of the parameters in NSGA II., Table S1: The information of water molecules for the data sets, Table S2: The best RMSD values for the recruit of the ligands and the computational time in the multi-body docking simulations considering the key water molecules as variable-length optimization variables, Table S3: Results of the multi-body docking simulations considering the key water molecules as fixed-length optimization variables and variable-length optimization variables.
