# Supplementary material for: Multi-Body Interactions in Molecular Docking Program Devised with Key Water Molecules in Protein Binding Sites
Source: Molecules. 2018 Sep 11;23(9):2321. doi: 10.3390/molecules23092321 (PMC6225211; doi:10.3390/molecules23092321)
Supplement: Supplementary file 1 [file molecules-23-02321-s001.pdf]

Multi-body Interactions in Molecular Docking Program Devised with Key Water Molecules in Protein Binding Sites

Wei Xiao [1,2], Disha Wang [2], Zihao Shen [2], Shiliang Li [2] and Honglin Li [1,2,*]

[1] School of Information Science and Engineering, East China University of Science and Technology, 130 Meilong Road, Shanghai 200237, China; xiaowei1012163@163.com

[2] Shanghai Key Laboratory of New Drug Design, School of Pharmacy, East China University of Science and Technology, 130 Meilong Road, Shanghai 200237, China; dishawang@foxmail.com (D.W.); saika_mx@163.com (Z.S.); slli403@163.com (S.L.)

* Correspondence: hlli@ecust.edu.cn; Tel.: +86-21-642-50213

Appendix A. Comparison of the parameters in NSGA II.

The parameters have great influence on the performance of the optimization algorithm. Different parameter combinations reflect different search capabilities of obtaining the optimal solutions in optimization algorithm. Therefore, a set of the key parameters of the Non-dominated Sorting Genetic Algorithm II (NSGA II) were compared in sequence, which included the population size, the evolutionary generation and the mutation probability. Take the crystal structure 2QU9, the crystal structure of the complex of group II phospholipase A2 with Eugenol, as an example.

Population Size. The population sizes were respectively set to 100, 500, 1000, 2000, and 3000 to determine its effect on the evolution. Three evaluation factors were simultaneously considered in this study: the relationships between the two object function values and the generations, the sizes of the optimal solutions, and the distributions of the Pareto-optimal solutions. From the factor of the relationships between the two object function values and the generations of crystal structure 2QU9 in Figure S1, the blue, green, red, cyan, magenta and yellow curves represented the minimum value, one quarter value, the intermediate value, three quarters value, the maximum value and the average value of the object function values in each generation during the evolution, respectively. At the beginning of the evolution, the object function values changed greatly in the populations with different population sizes. However, at the end of the evolution, the one quarter value, the intermediate value, three quarters value of all the object function values roughly reached a stable state, while there was a fluctuation in the population with the population size 3000. These results illustrated that the populations with the population sizes 100 to 2000 converged to a stable state, but it was hard to get convergence with an oversized population size 3000 at the end of the evolution. Furthermore, the difference values of the object function values, in terms of the values between the maximum values and the minimum values of the object functions, in the population with the population size 1000 or 2000 were greater than those in the population with the population size 100 or 500 at the end of the evolution. This suggested that the diversity of the population was higher and the ability of obtaining the global optimal solutions was greater in the population with the population size 1000 or 2000. While the difference values of the object function values in the population with the oversize population size 3000 were also minor. It was speculated that the population may lead to a local optimum. In addition, from the other factor of the sizes of the optimal solutions (Figure S2), the sizes of the optimal solutions presented an approximate level in the population with the population sizes 1000 to 3000. While the size of the optimal solutions was lower at the end of the evolution when the population size was set to 100 or 500. And besides, from the distribution of the

Pareto-optimal solutions, the Pareto-optimal solutions in the five schemes were in front of the solution to the pose of the crystal structure (the red dots in Figure S3). These results suggested that the populations evolved in an optimized direction. Especially, the distribution of the Pareto-optimal solutions in the population with the population size 2000 was more uniform and extensive than that in the population with the population size 1000. Given the above, the population size 2000 was chosen as the reference value when using this method.

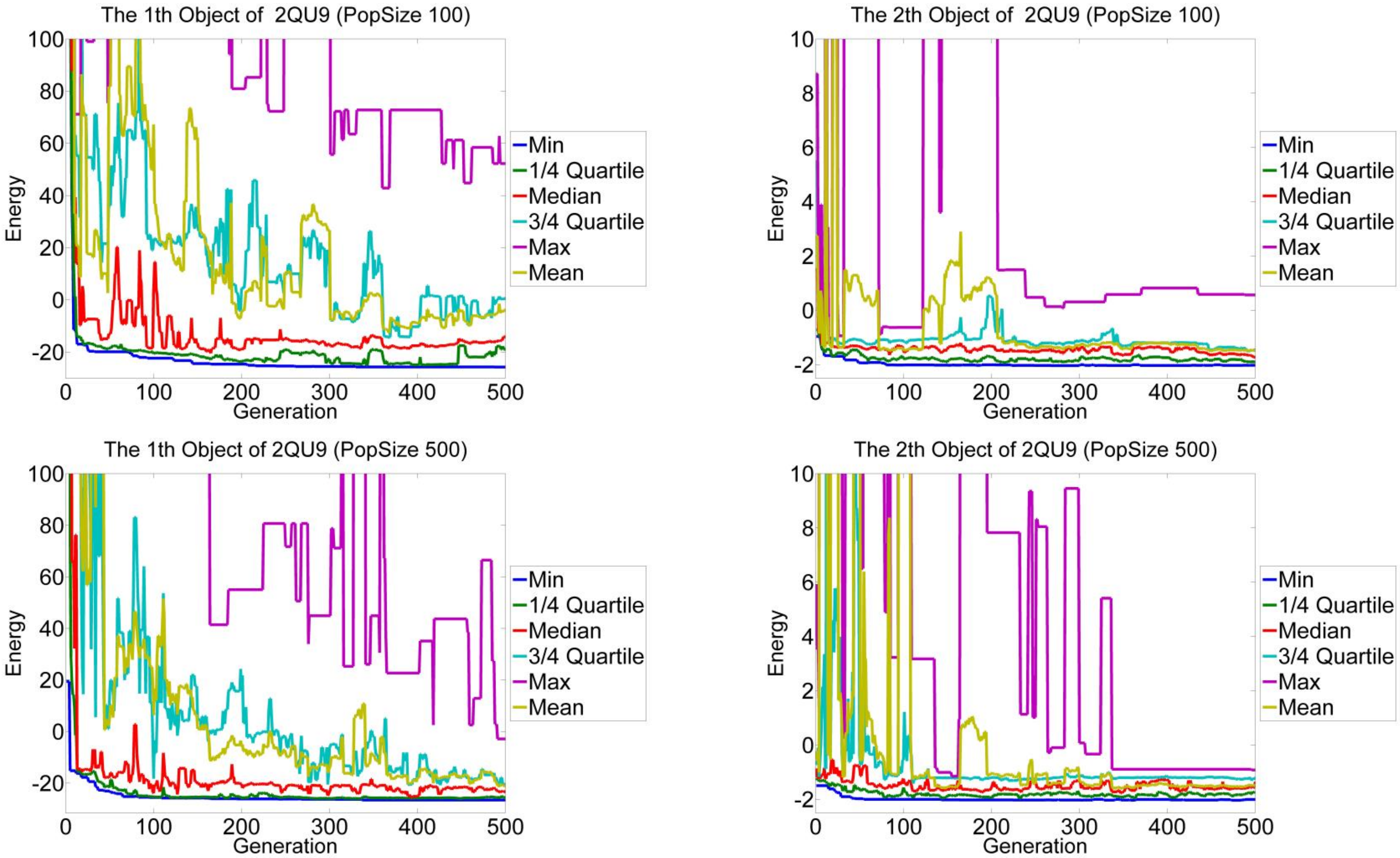

Figure S1. The relationships between the object function values and the generations of crystal structure 2QU9 with different population sizes.

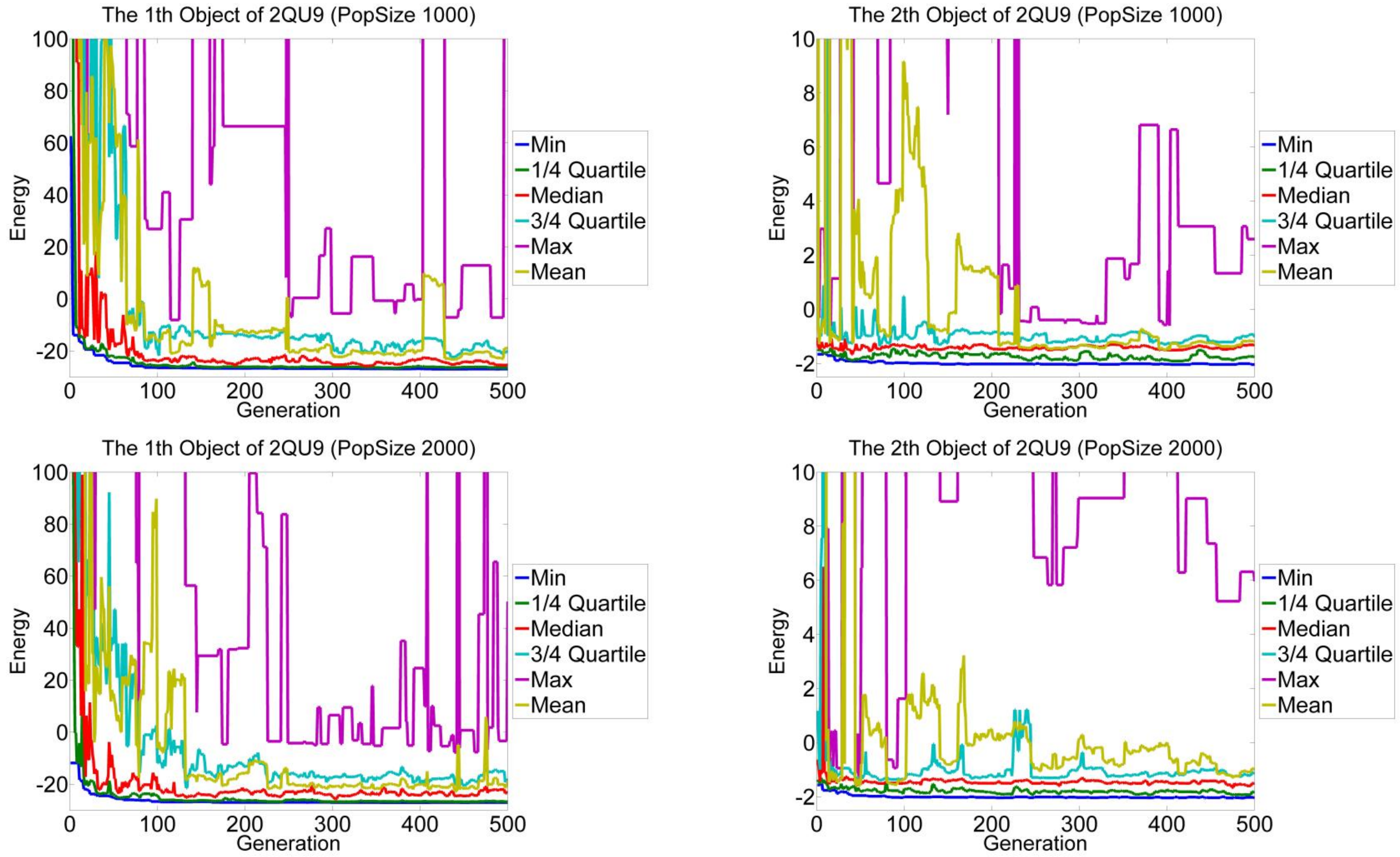

Figure S1. (continue).

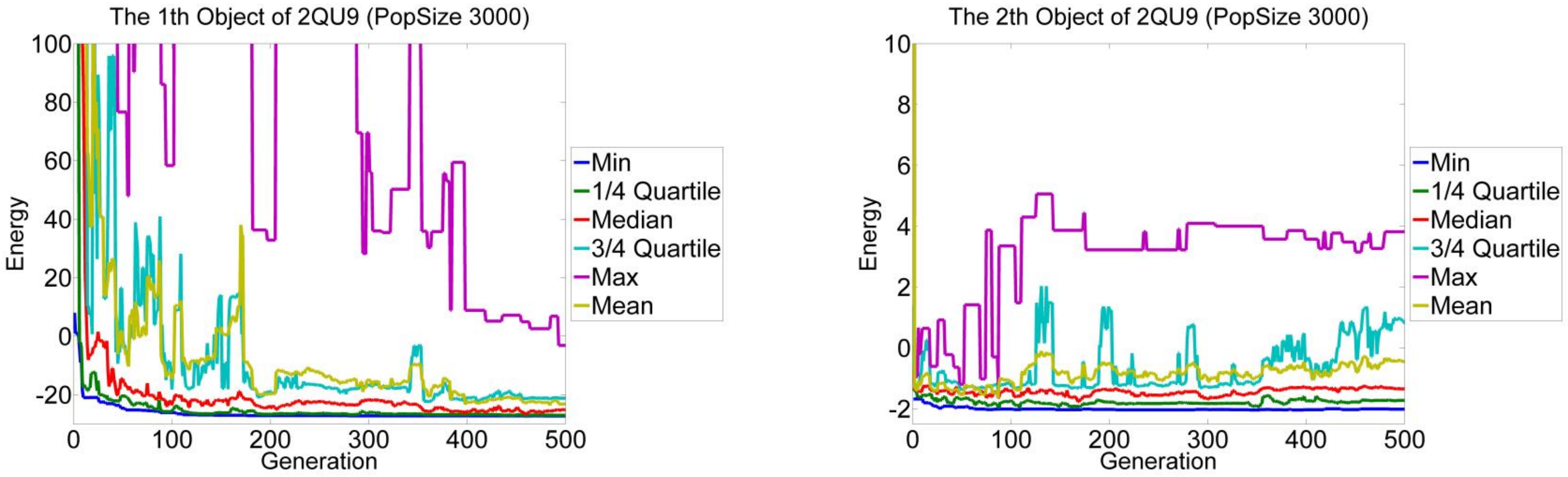

Figure S1. (continue).

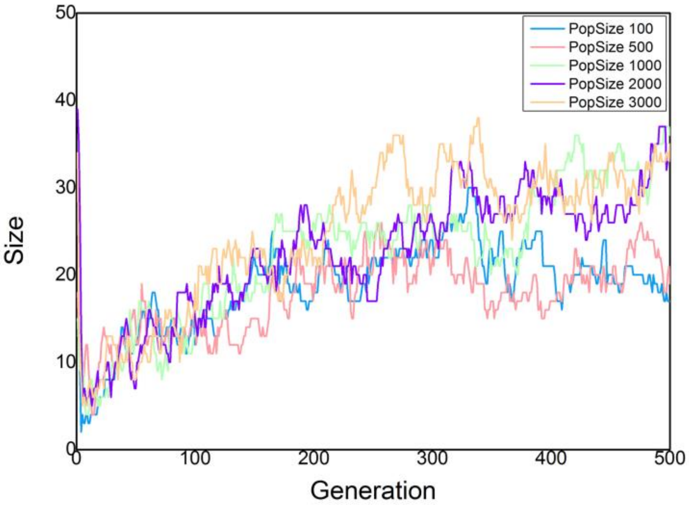

Figure S2. The sizes of the optimal solutions in optimization of crystal structure 2QU9 with different population sizes.

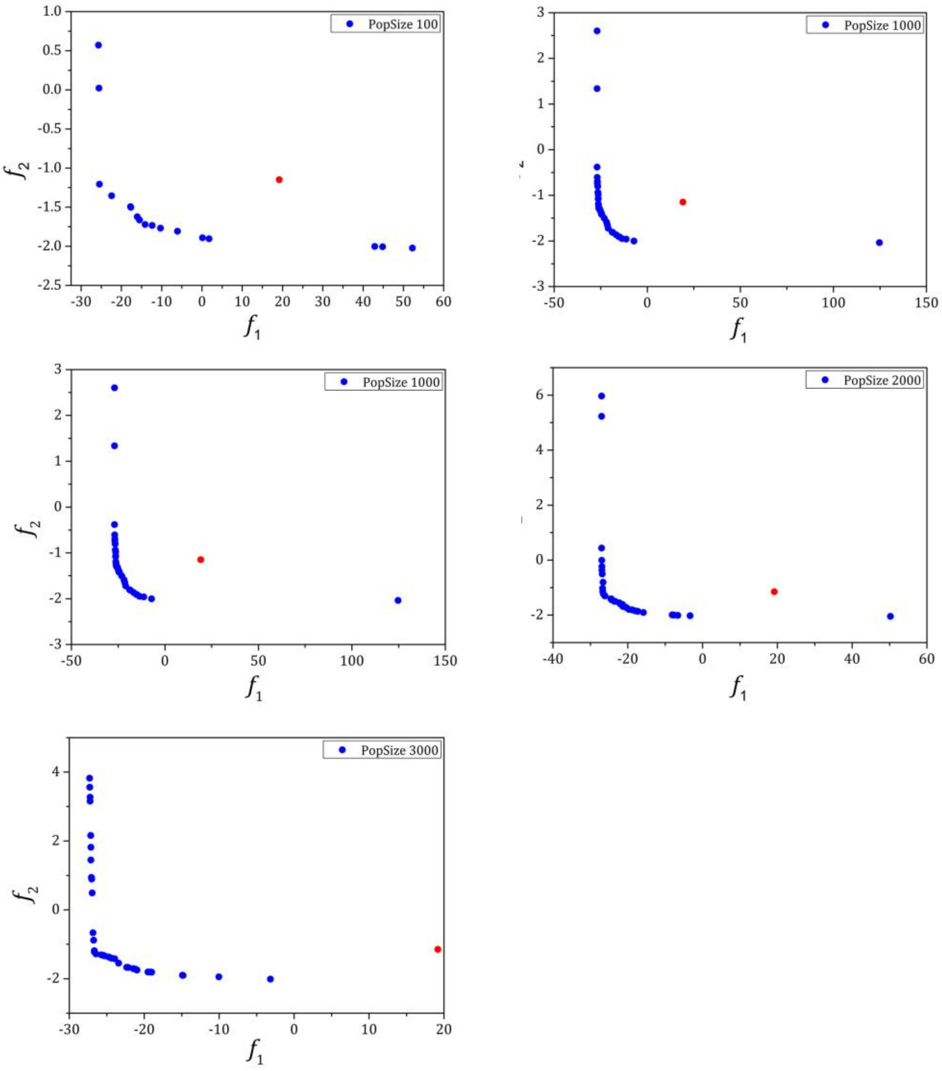

Figure S3. The distribution of the Pareto-optimal solutions in optimization results of crystal structure 2QU9 with different population sizes.

Evolutionary Generation. The evolutionary generations were respectively set to 500 and 1000 to determine its effect on the evolution from the same evaluation factors for the population sizes. According to the relationships between the two object functions values and the generations (Figure S4), the one quarter value, the intermediate value, three quarters value of all the object function values obtained a relatively stable state in 400 to 500 generations and presented no obvious difference after 500 generations. It showed that the population achieved greater convergence within 400 to 500 generations. However, excessive evolutionary generations greatly increased the computational cost and reduced the execution efficiency of the evolution. Furthermore, on the basis of the sizes of the optimal solutions (Figure S5) and the distribution of the Pareto-optimal solutions (Figure S6), using both of the evolutionary generations could obtain a reasonable size and uniformly distributed solutions. Considered the above factors, the value of 500 was chosen as the reference value for the evolutionary generation.

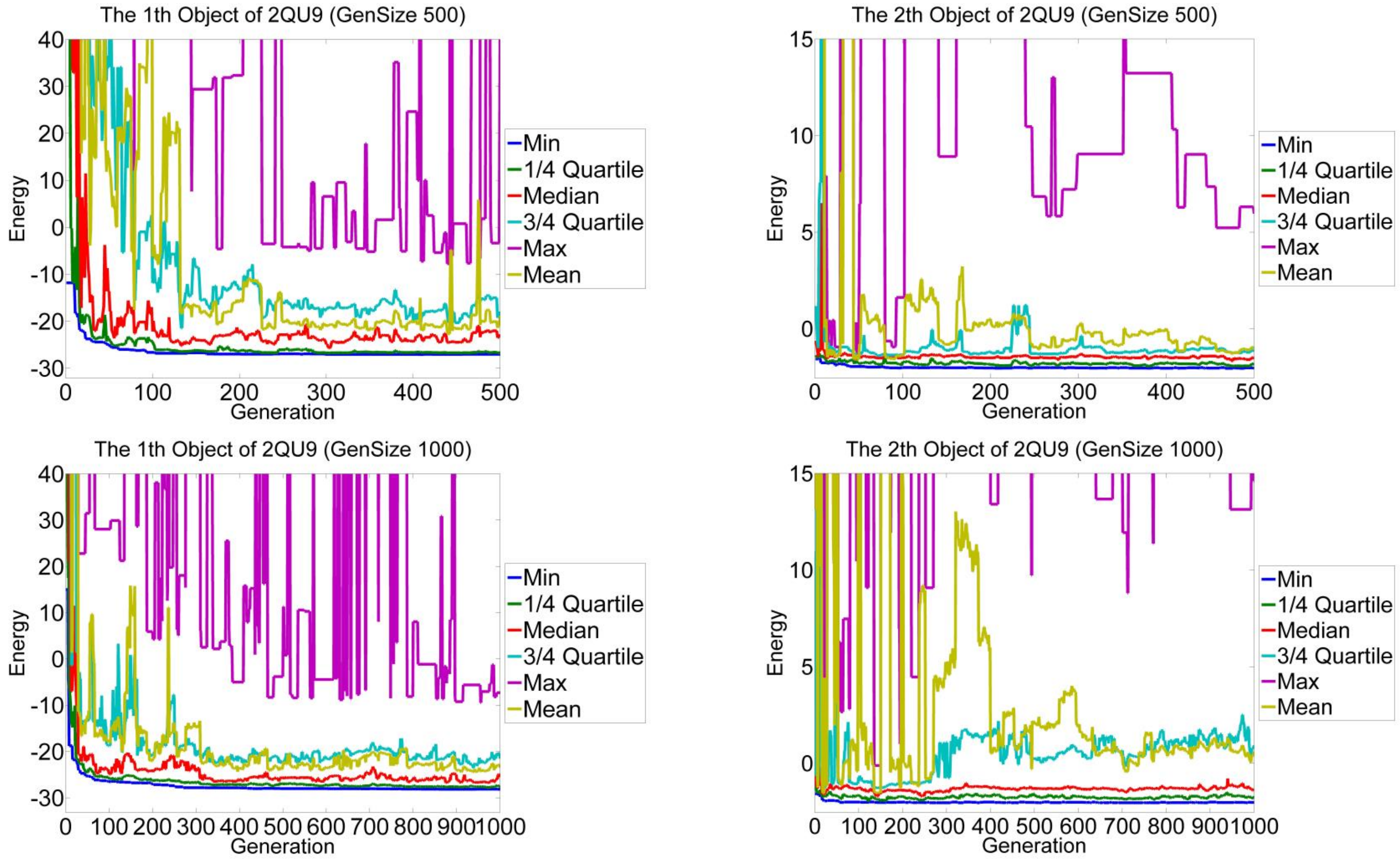

Figure S4. The relationships between the object function values and the generations of crystal structure 2QU9 with different generations.

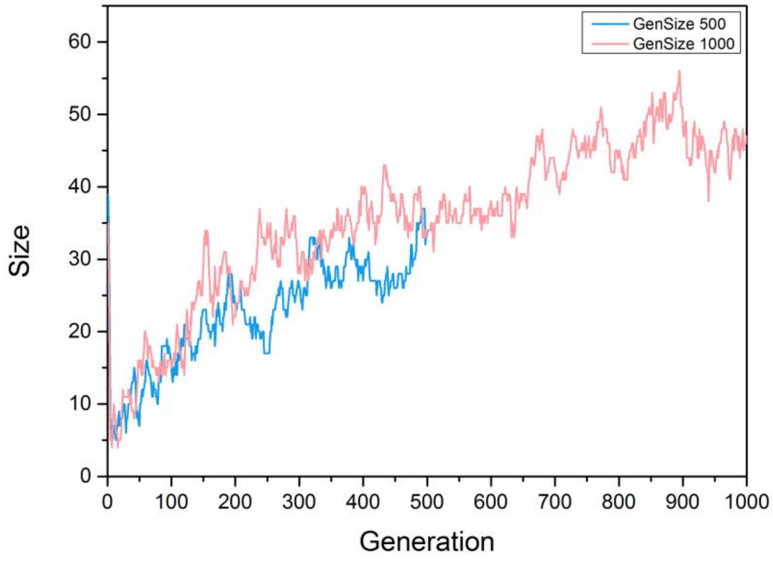

Figure S5. The sizes of the optimal solutions in optimization of crystal structure 2QU9 with different generations.

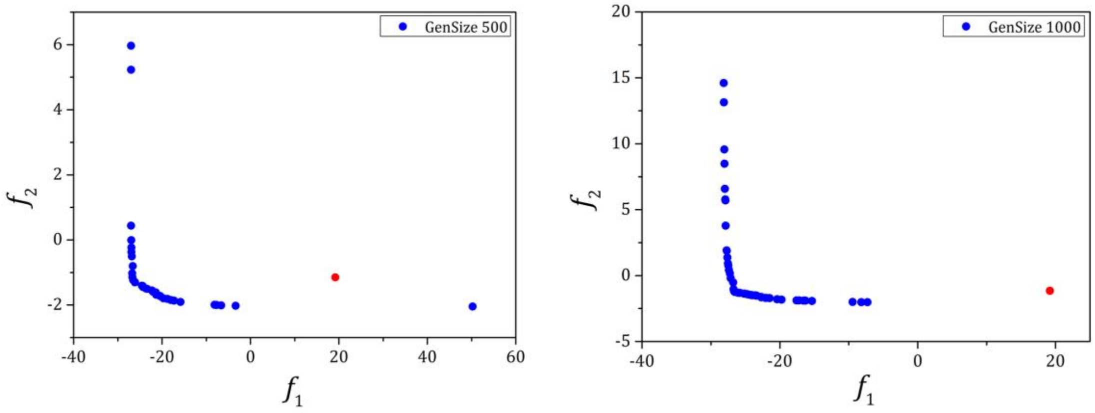

Figure S6. The distribution of the Pareto-optimal solutions in optimization results of crystal structure 2QU9 with different generations.

Mutation Probability. The mutation probabilities were set to 0.05, 0.10, 0.20, and 0.30 to perform the comparison in this study. When the mutation probability was 0.05, the maximum object function values would be close to the minimum object function values in the evolution (Figure S7). It may be that the difference values among the individuals were minor in the optimized population with a smaller mutation probability, which was not conducive to the diversity of the population and may lead to the prematurity of the population. However, by adopting a high mutation probability 0.20 or 0.30, the genetic algorithm would then degraded to a random search algorithm, which also made it difficult for the population to converge. In addition, from the sizes of the optimal solutions in Figure S8 and the distribution of the

Pareto-optimal solutions in Figure S9, all the schemes with different evolutionary generations could obtain a reasonable size and uniformly distributed solutions. Considering the above factors, the value of 0.10 was chosen as the reference value for the mutation probability.

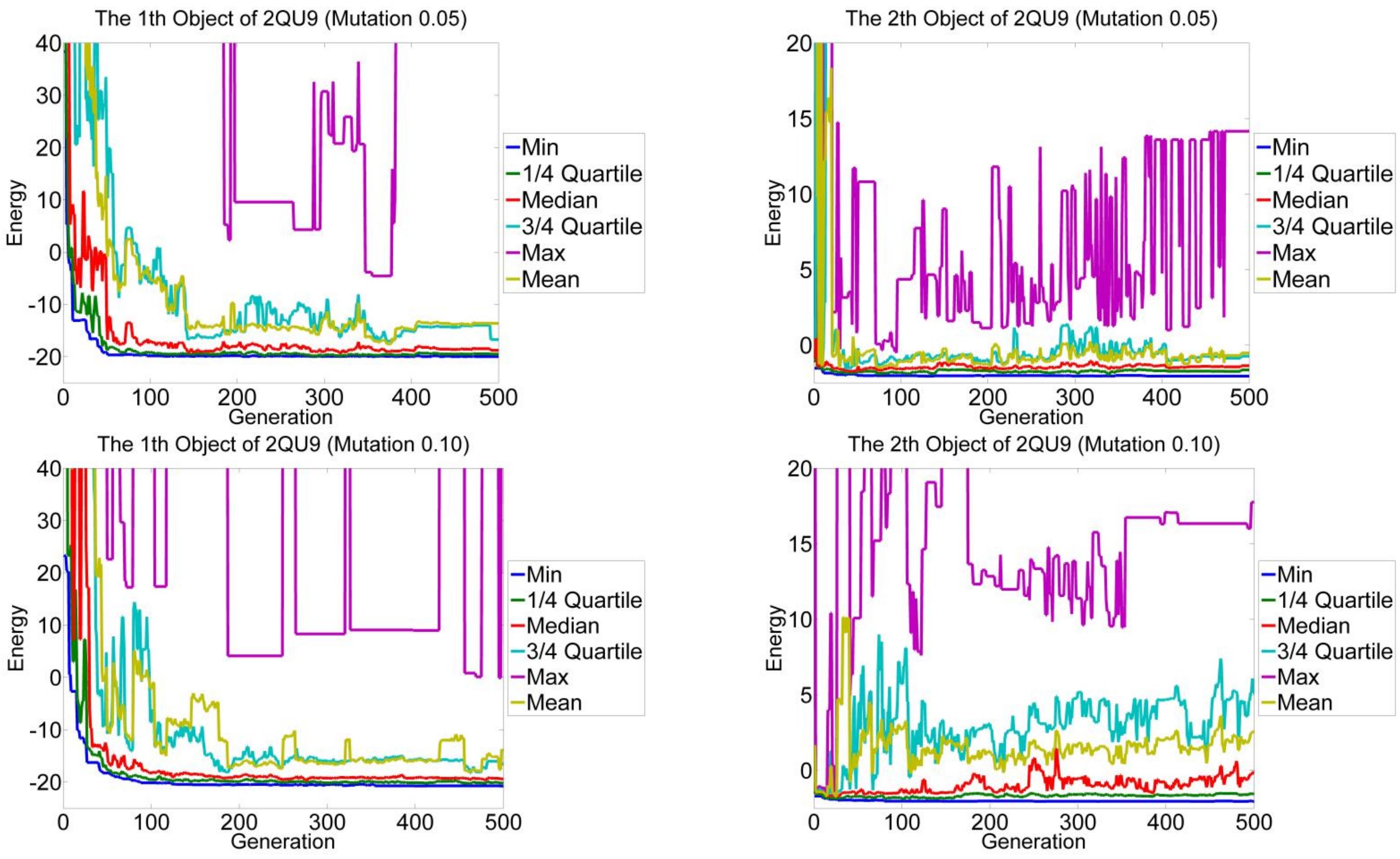

Figure S7. The relationships between the object function values and the generations of crystal structure 2QU9 with different mutation probabilities.

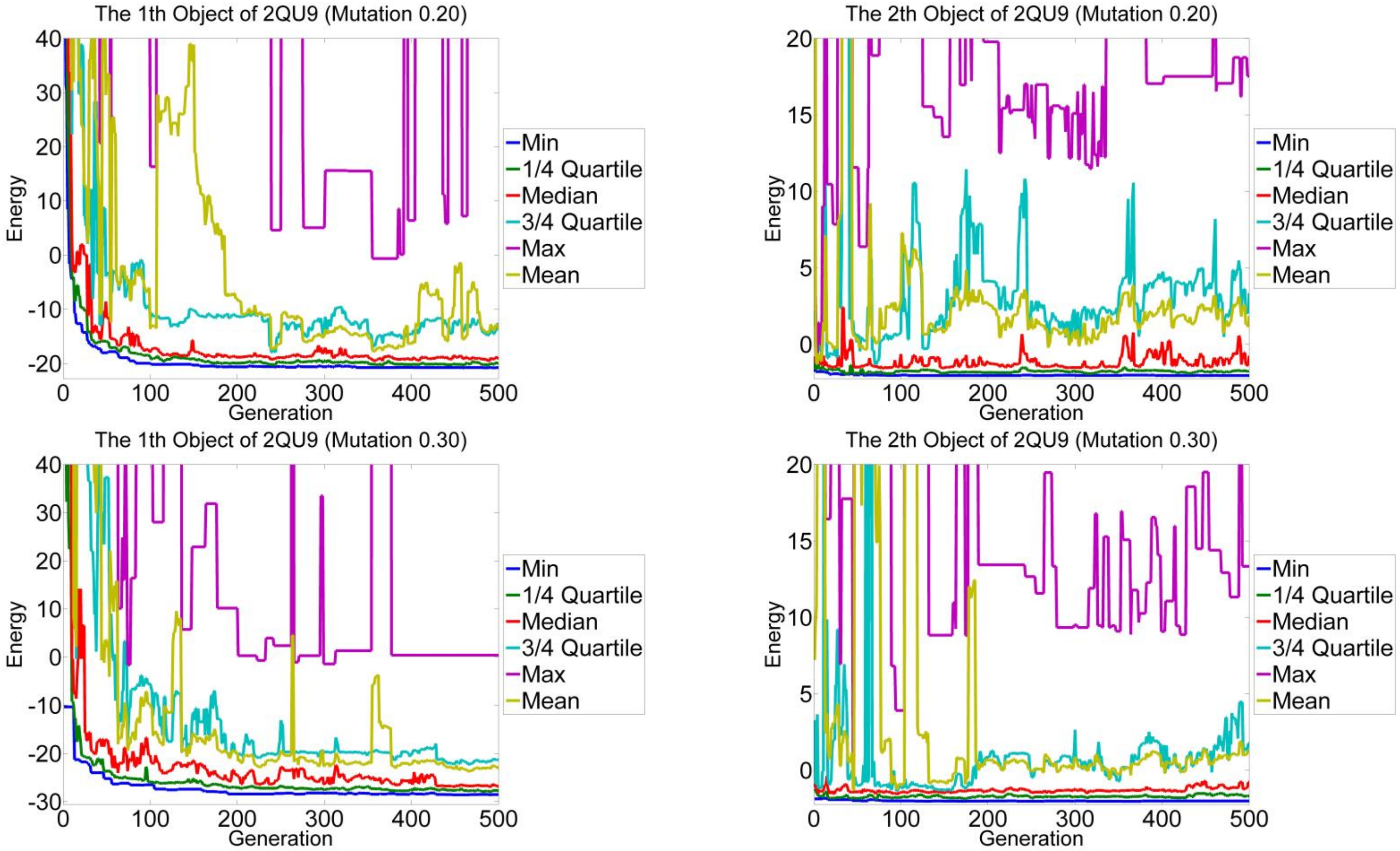

Figure S7. (continue).

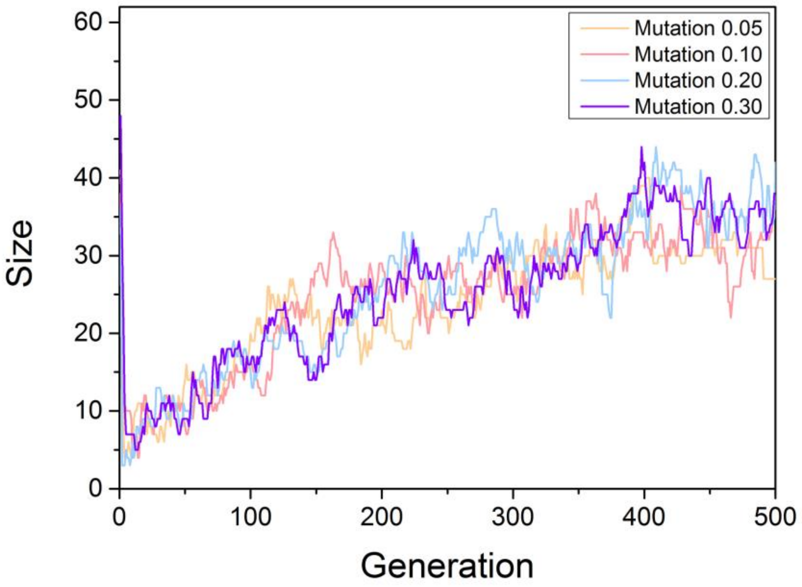

Figure S8. The archive sizes in optimization of crystal structure 2QU9 with different mutation probabilities.

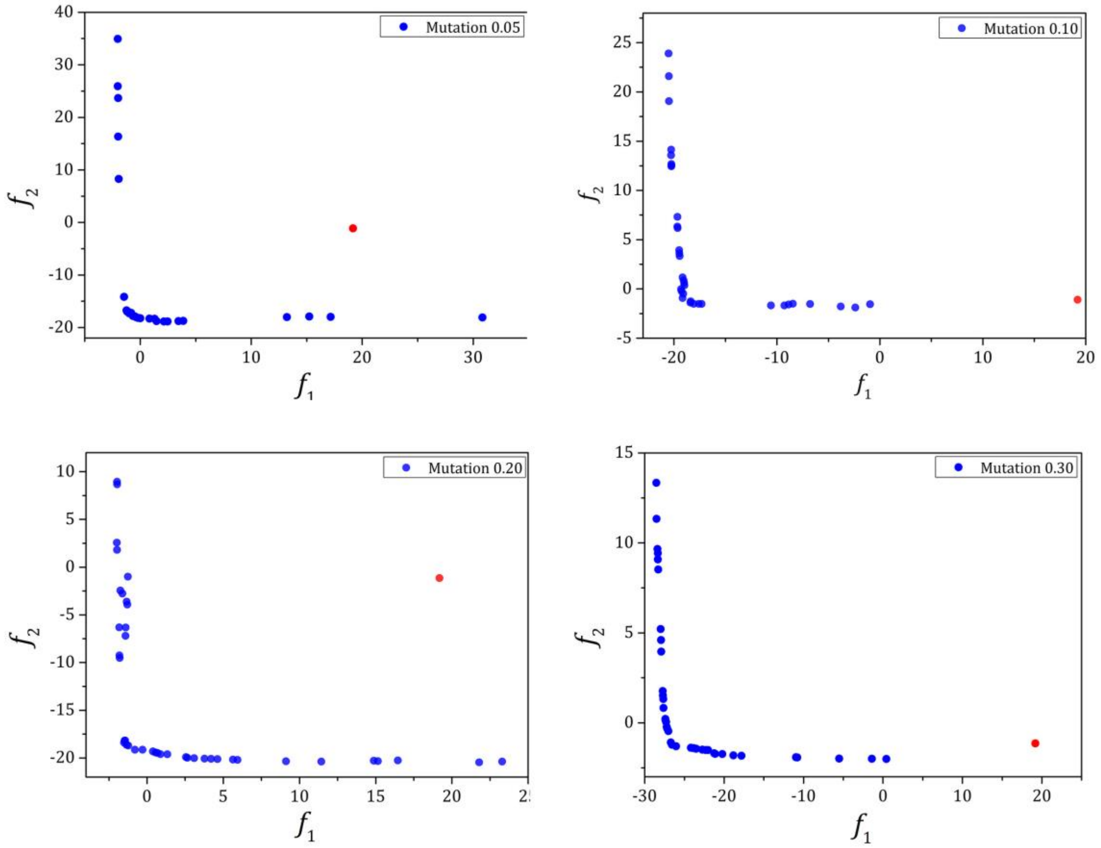

Figure S9. The distribution of the Pareto-optimal solutions in optimization results of crystal structure 2QU9 with different mutation probabilities.

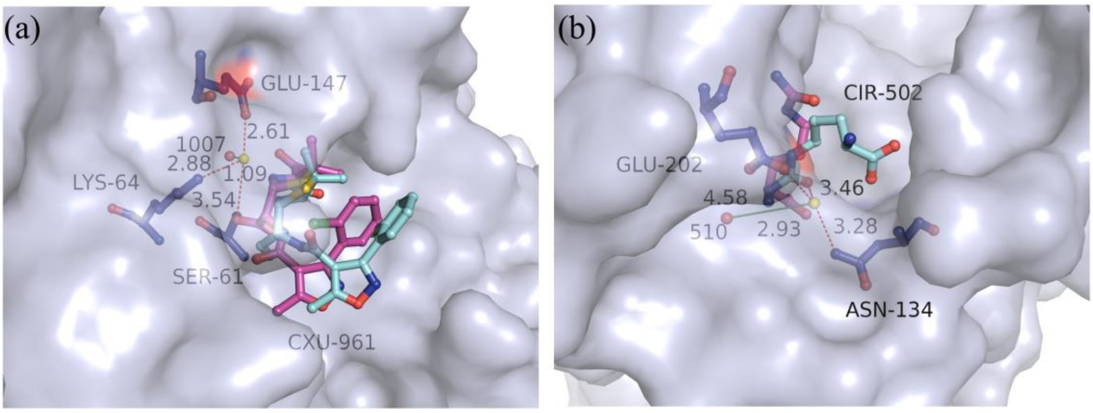

Figure S10. The docked poses of the best RMSD values for the recruit of the ligands in the multi-body docking program considering the key water molecules as fixed optimization variables of crystal structure (a) 1FCM and (b) 1K97. The X-ray poses and the docked poses of the ligands are shown as magenta and cyan ball-and-stick models, respectively. The yellow and red spheres represent the optimized water molecules and the experiment-determined water molecules, respectively. The hydrogen bonds between the water molecules and the amino acid residues or the ligands are represented by red dashed lines. The distances between the two water molecules are represented by green solid lines. Numbers beside the lines are the lengths.

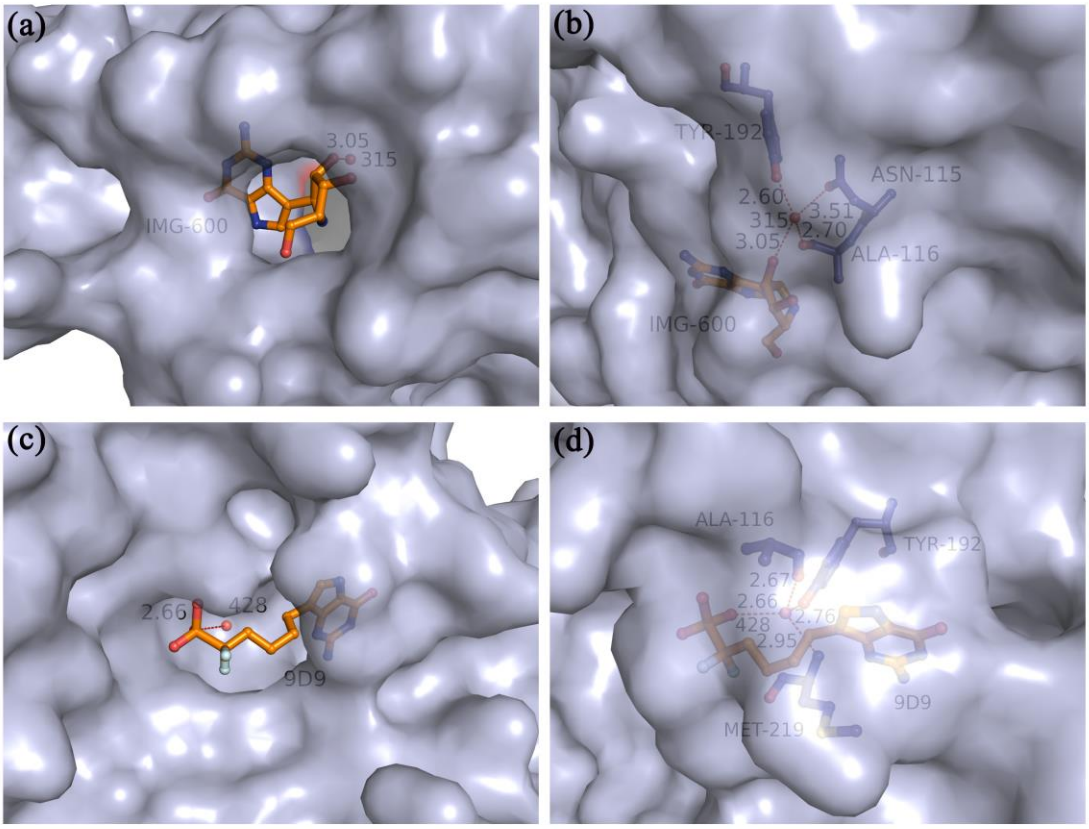

Figure S11. The poses of crystal structures 1B8N (a-b) and 3FUC (c-d). The X-ray pose is shown as orange ball-and-stick models. The experiment-determined water molecule W428 is represented as red sphere. The hydrogen bonds between the water molecules and the nearby amino acid residues (blue ball-and-stick model) or the ligands (orange ball-and-stick model) are represented by red dashed lines. Numbers beside the dashed lines are the lengths.

Table S1. PDB codes for the data sets

PDB Code	the sites of the water molecules in the X-ray structures	the predicted hydration sites by tetrahedron-water-cluster model	Distances (Å)	Numbers of water moleculesb
1A8I	(31.837 26.280 26.914)	(33.533 26.113 25.576)	2.17	37
1CB0	(57.011 20.800 24.992)	(57.588 20.249 24.179)	1.14	7
1D2A	(-1.056 63.253 25.83)	(0.053 63.927 25.344)	1.39	8
1DCP	(46.649 32.235 41.777)	(46.493 32.754 41.826)	0.54	5
1E2P	(15.464 25.643 10.107)	(14.922 25.389 11.360)	1.39	6
1FCM	(74.064 6.021 26.612)	(73.027 5.779 26.076)	1.19	17
1FPU	(19.078 53.053 15.846)	(19.788 52.346 15.674)	1.02	11
1G2O	(55.447 49.68 40.797)	(55.019 48.614 40.943)	1.16	6
1G4K	(18.010 28.075 52.026)	(18.049 28.893 53.293)	1.51	7
1H0C	(29.363 31.125 -7.586)	(28.497 32.425 -7.407)	1.57	8
1H0S	(26.039 73.763 15.481)	(26.026 72.284 15.487)	1.48	10
1I80	(0.715 16.081 8.466)	(1.410 17.283 8.335)	1.39	7
1IFS	(56.649 -24.791 93.889)	(56.639 -23.087 94.921)	1.99	7
1K7E	(46.179 31.954 8.783)	(47.584 34.065 11.868)	3.99	7
1K97	(39.626 18.579 45.38)	(38.638 18.105 44.370)	1.49	16
1MQH	(13.01 47.781 15.083)	(12.962 48.380 16.311)	1.37	15
1OWE	(19.245 24.168 35.675)	(18.539 23.296 35.431)	1.15	15
1OWH	(19.208 24.170 35.554)	(17.84 22.870 35.243)	1.91	10
1SJ0	(22.408 0.102 25.689)	(23.410 -0.583 24.164)	1.95	11
1SPA	(21.833 10.076 -14.657)	(20.097 9.492 -14.986)	1.86	9
1SQO	(18.955 24.025 35.816)	(18.370 23.411 35.691)	0.86	8
1SYI	(14.361 12.720 91.928)	(13.933 13.703 91.924)	1.07	10
1T46	(27.019 27.783 41.340)	(28.195 28.118 40.509)	1.48	23
1TNJ	(32.142 13.950 10.624)	(31.214 13.165 11.479)	1.49	8
1TX2	(-64.589 55.810 141.639)	(-64.710 55.116 140.762)	1.12	10
1WBU	(3.415 3.128 32.897)	(3.497 4.243 32.024)	1.42	12
1XM6	(48.845 10.621 -22.032)	(49.716 12.122 -21.402)	1.85	27
1YPE	(7.070 8.826 -0.321)	(6.646 9.019 0.769)	1.19	17
1ZEH	(2.052 14.578 7.135)	(2.410 16.020 7.795)	1.63	10
1ZP5	(0.919 28.496 11.758)	(2.869 28.383 10.453)	2.35	15
1ZUI	(39.607 6.654 -0.510)	(39.572 7.834 -1.106)	1.32	9
2AXA	(26.617 1.209 13.460)	(25.418 0.799 12.941)	1.37	5
2B7N	(10.414 137.138 -2.995)	(9.233 136.712 -2.415)	1.38	4
2DHN	(45.855 49.095 73.903)	(46.195 49.401 74.548)	0.79	2
2F7X	(14.097 7.102 1.087)	(13.621 7.625 1.948)	1.11	19
2G72	(26.347 48.728 -28.217)	(27.403 50.288 -28.414)	1.89	9
2IYR	(-19.484 2.045 48.117)	(-19.850 2.444 48.071)	0.54	9
2OIQ	(-0.303 -35.691 8.659)	(-0.215 -35.489 9.387)	0.76	11

2OJJ	(-10.449	8.674	38.527)	(-9.870	9.575	38.235)	1.11	13
2QTT	(-2.992	-1.134	-29.094)	(-2.639	-1.609	-29.472)	0.70	11
2QU9	(8.587	29.148	-7.044)	(8.022	28.575	-6.018)	1.30	3
2R3H	(3.428	25.024	9.099)	(3.212	24.405	9.021)	0.66	14
2SLI	(6.333	10.970	38.109)	(5.703	9.943	38.265)	1.21	18
2UY4	(41.496	22.401	18.375)	(41.460	22.793	18.124)	0.47	13
2VBW	(-0.074	14.225	1.050)	(-0.435	12.194	4.152)	3.73	3
2VCQ	(-30.861	8.194	14.603)	(-30.840	8.328	14.833)	0.27	14
2VDJ	(-26.751	0.161	7.460)	(-26.613	-0.700	6.897)	1.04	17
2VK6	(4.123	31.580	17.703)	(4.016	32.888	18.679)	1.64	14
2VVZ	(21.424	1.913	97.241)	(22.062	0.913	96.510)	1.39	2
2W6O	(20.585	13.251	1.453)	(20.687	14.915	0.400)	1.97	4
2W6P	(-0.455	-20.931	27.175)	(-0.865	-21.341	27.325)	0.60	11
2X8D	(8.365	-3.287	16.400)	(8.895	-3.520	16.655)	0.63	16
2XDA	(5.329	15.765	21.514)	(5.654	16.131	22.115)	0.78	9
2XK8	(21.525	6.663	-10.338)	(20.938	7.390	-11.883)	1.81	8
2YIG	(23.553	17.281	9.023)	(23.762	15.536	9.624)	1.86	21
2ZPU	(11.691	-6.244	17.820)	(11.101	-6.140	17.938)	0.61	12
3BE2	(38.619	34.999	12.858)	(38.664	34.797	13.375)	0.56	22
3BMY	(1.136	4.277	12.122)	(1.297	4.641	12.215)	0.41	14
3C84	(3.930	-20.813	63.908)	(4.660	-20.762	64.048)	0.85	12
3D14	(-11.411	26.691	73.721)	(-11.821	26.814	74.150)	0.61	16
3D6O	(31.936	-6.792	11.472)	(30.905	-6.098	10.247)	1.75	19
3EBO	(31.238	24.468	24.875)	(31.254	24.829	26.818)	1.98	33
3EHY	(-0.667	1.178	-5.380)	(0.665	0.519	-6.158)	1.68	13
3F0T	(15.363	91.948	43.392)	(15.496	91.559	43.373)	0.41	8
3FDZ	(19.347	-36.311	59.427)	(20.188	-35.867	59.829)	1.03	9
3FMH	(19.256	15.617	30.713)	(19.140	15.772	31.147)	0.48	13
3FTU	(28.681	-0.963	5.056)	(28.342	-0.566	4.085)	1.10	11
3GCQ	(-2.326	-6.674	-17.360)	(-2.652	-5.862	-17.116)	0.91	15
3FUH	(35.226	75.409	-1.479)	(36.589	76.347	-0.782)	1.80	33
3G4K	(-14.264	2.710	29.083)	(-14.967	1.387	29.426)	1.54	47
3GQZ	(-30.652	13.335	23.495)	(-30.040	14.171	23.247)	1.07	19
3HMM	(11.410	66.269	7.724)	(11.492	65.726	8.793)	1.20	9
3HV7	(-2.038	-6.932	-17.348)	(-2.554	-5.839	-16.360)	1.56	6
3KBZ	(32.991	0.913	38.293)	(32.342	1.169	37.998)	0.76	5
3KCU	(54.932	28.468	135.219)	(54.090	28.119	136.652)	1.70	4
3KJD	(-7.304	34.600	35.575)	(-6.700	34.618	34.432)	1.29	19
3L5T	(16.993	-2.808	5.247)	(18.493	-2.729	6.176)	1.77	2
3LE8	(17.501	14.521	3.123)	(16.598	13.482	3.743)	1.51	52
3OZD	(44.205	29.647	-16.831)	(43.925	31.555	-16.141)	2.05	4
3PD9	(53.101	25.620	44.272)	(54.223	25.249	44.626)	1.23	8
3QVK	(17.157	17.120	8.692)	(17.294	16.400	7.362)	1.52	7
3RXJ	(-4.749	-15.139	22.977)	(-4.090	-15.007	22.086)	1.12	13

3SHC	(6.807	8.461	-0.081)	(6.446	8.581	0.765)	0.93	18
3SKH	(24.964	4.445	-1.448)	(25.331	4.037	-0.378)	1.20	11
3SLI	(6.286	11.173	38.278)	(5.572	9.980	38.374)	1.39	17
3SMB	(37.706	12.031	7.755)	(38.075	11.669	8.827)	1.19	4
3T1M	(23.056	-9.427	1.479)	(23.376	-9.049	2.662)	1.28	18
3UPN	(12.605	-62.768	10.883)	(12.160	-63.504	11.769)	1.23	2
3ZYA	(14.069	-10.901	-29.977)	(14.847	-9.829	-29.581)	1.38	14
4AQH	(-27.331	2.857	3.319)	(-27.325	2.713	3.486)	0.22	3
4B6O	(-47.608	-25.295	-9.920)	(-47.777	-24.994	-9.108)	0.88	11
4B6P	(25.500	47.576	9.909)	(25.111	47.829	9.226)	0.83	7
4B6R	(16.197	28.795	-5.175)	(15.559	29.025	-5.244)	0.68	9
4FU8	(-19.178	-24.198	-5.264)	(-18.525	-23.370	-5.345)	1.06	16
4FUH	(-8.411	-24.477	5.372)	(-9.426	-23.338	5.585)	1.54	25
4G8Y	(32.469	7.092	24.929)	(31.313	6.376	26.065)	1.77	21
4GJ3	(-4.926	-0.028	-14.676)	(-4.886	-0.051	-13.191)	1.49	6
4IFV	(46.955	-1.741	23.849)	(46.371	-3.143	24.731)	1.76	16
4CSV	(-18.302	10.410	-0.395)	(-17.946	10.621	-0.737)	0.54	5
4WKN	(10.734	44.730	-31.380)	(10.032	45.554	-31.217)	1.09	3
4THI	(12.856	25.704	3.153)	(12.945	26.238	2.643)	0.74	10
4ZHM	(-0.678	-40.101	-29.479)	(-0.506	-39.691	-28.772)	0.84	3
5IUI	(66.944	73.793	9.562)	(66.756	74.646	8.709)	1.22	6

[a] Distances between the predicted hydration sites by the tetrahedron-water-cluster model and the location of the water molecules in the X-ray structures

[b] Number of water molecules in the binding sites

Table S2. The best RMSD values for the recruit of the ligands and computational time in the multi-body docking simulations considering the key water molecules as variable optimization variables

N_{rot} [a]	N_{com} [b]	min RMSD (Å)	max RMSD (Å)	avg RMSD (Å)	min Time (s)	max Time (s)	avg Time (s)
1-5	49	0.15	5.68	1.54	59.21	578.13	167.85
6-10	42	0.18	3.65	1.57	64.72	527.14	220.18
11-15	12	0.14	4.46	1.71	107.19	538.41	291.25
all	103	0.14	5.68	1.57	59.21	578.13	203.57

[a] Number of rotatable bonds of the ligands
[b] Number of the complexes

Table S3. Results of the multi-body docking simulations considering the key water molecules as fixed optimization variables and variable optimization variables

PDB code	The multi-body docking simulations considering the key water molecules as fixed optimization variables				The multi-body docking simulations considering the key water molecules as variable optimization variables			
	The best RMSD values for the recruit of the ligands (Å)	Scores(KJ/mol)	The numbers of the water molecules	The RMSD values of the water (Å)	The best RMSD values for the recruit of the ligands (Å)	Scores(KJ/mol)	The numbers of the water molecules	The distances of the nearest water molecules(Å)
1A8I	0.68	-33.08	1	0.62	0.14	-59.6	3	1.21/0.59/1.27
1CB0	0.41	-3.02	1	0.76	0.15	-36.13	2	3.51/1.78
1D2A	1.86	-21.01	1	1.80	1.10	0.41	3	1.63/1.45/2.52
1DCP	1.33	-33.28	1	1.21	1.10	-42.68	3	4.10/1.81/7.36
1E2P	1.95	-36.91	1	0.73	0.45	-58.06	3	2.99/2.99/3.46
1FCM	2.36	-40.28	1	1.19	2.46	-44.55	1	3.63
1FPU	0.47	-56.94	1	1.86	1.91	-28.19	3	4.45/3.51/3.32
1G2O	0.29	-47.00	1	0.51	0.29	-56.51	2	4.11/1.99
1G4K	0.34	-38.51	1	2.48	0.27	-49.49	1	6.76
1H0C	3.99	-7.93	1	4.06	3.90	-26.36	2	2.77/6.36
1H0S	1.71	-44.67	1	2.91	1.66	-57.00	3	2.31/0.56/1.69
1I80	0.38	-30.94	1	1.33	0.35	-30.65	1	6.72
1IFS	0.53	-24.78	1	1.37	0.54	-32.68	2	1.76/0.95
1K7E	1.41	2.46	1	1.22	0.91	-46.69	1	1.99
1K97	5.42	-37.16	1	4.58	1.86	-40.40	1	1.83
1MQH	0.34	-51.37	1	0.54	0.81	-20.63	1	1.53
1OWE	0.64	-38.73	1	2.35	1.32	-44.32	2	2.74/2.79
1OWH	0.82	-40.78	1	2.42	1.02	-43.91	1	2.92
1SPA	1.82	-18.46	1	0.70	1.85	142.76	3	0.42/4.26/2.60

1SQO	0.78	-41.89	1	2.10	0.79	-19.36	2	3.16/4.79
1SJ0	1.86	-56.67	1	1.67	1.80	51.18	2	0.89/0.25
1SYI	0.21	-40.47	1	0.37	1.38	-19.27	1	1.12
1T46	0.88	-24.97	1	0.29	0.59	-56.40	2	0.57/0.36
1TNJ	2.96	-10.56	1	3.86	2.89	-27.57	2	4.17/0.37
1TX2	1.61	-7.53	1	2.70	3.65	-27.86	3	2.75/2.72/1.83
1WBU	0.62	-15.04	1	1.09	1.16	-30.78	3	1.96/1.57/2.44
1XM6	1.25	-41.04	1	0.33	0.14	-46.15	2	4.69/1.99
1YPE	1.36	-15.26	1	1.24	1.77	-40.15	3	4.89/5.18/0.75
1ZEH	2.57	-20.20	1	1.79	1.36	-7.76	3	2.46/3.51/0.31
1ZP5	2.66	-22.10	1	2.08	1.89	-31.08	1	1.03
1ZUI	0.35	-32.29	1	0.28	1.46	-12.89	2	0.42/1.00
2AXA	1.00	-21.11	1	2.24	1.37	49.21	2	0.57/1.22
2B7N	0.74	-15.41	1	1.72	0.89	-17.67	2	1.59/4.20
2DHN	2.42	-27.67	1	1.70	1.72	-34.29	3	1.39/5.01/4.69
2F7X	1.89	185.54	1	3.98	1.48	-46.94	1	1.45
2G72	0.22	-51.57	1	2.41	0.17	-51.26	2	5.18/0.91
2IYR	2.55	-15.42	1	1.18	2.60	-44.99	2	3.13/2.71
2OIQ	1.04	-67.42	1	2.59	2.55	6.29	1	11.22
2OJJ	1.52	-48.43	1	1.07	1.56	107.42	2	0.90/2.56
2QTT	0.85	-6.76	1	2.86	2.37	-4.71	3	3.62/1.83/2.45
2QU9	0.71	-27.69	1	2.25	1.07	-32.73	2	3.14/2.27
2R3H	1.08	-39.05	1	1.19	0.66	-47.47	3	1.56/0.48/1.86
2SLI	1.96	-39.04	1	2.84	0.77	-54.26	3	3.50/3.29/1.72
2UY4	1.24	-31.30	1	1.76	1.22	-36.73	3	4.85/1.80/3.39
2VBW	3.08	2.26	1	2.32	1.85	-16.04	2	2.54/6.19

2VCQ	1.44	-10.74	1	0.29	1.76	-53.64	1	0.53
2VDJ	0.87	-30.77	1	2.28	1.44	-40.11	3	4.56/2.24
2VK6	1.10	-51.67	1	0.39	1.10	-62.37	1	4.61
2VVZ	1.14	-47.98	1	0.43	0.99	-62.45	1	1.39
2W6O	0.61	-16.52	1	0.66	0.35	-5.93	2	0.41/3.05
2W6P	2.55	63.35	1	0.77	2.46	-12.45	2	3.53/5.10
2X8D	0.29	-32.14	1	1.61	0.22	-33.45	2	1.56/0.84
2XDA	4.84	-9.68	1	0.57	5.00	23.20	3	0.53/3.09/6.39
2XK8	2.55	-48.15	1	1.26	0.64	561.30	2	4.43/0.62
2YIG	1.39	-49.46	1	1.07	1.32	-32.78	3	0.50/2.15/1.83
2ZPU	1.59	-21.31	1	1.84	1.86	-16.28	1	2.90
3BE2	1.37	-31.06	1	1.81	3.92	602.42	2	1.24/2.17
3BMY	1.24	-39.28	1	1.76	1.73	-15.02	3	0.76/6.86/5.58
3C84	0.14	-46.82	1	0.77	0.16	-44.92	3	0.62/1.33/0.81
3D6O	1.33	-23.51	1	0.53	1.75	-40.41	3	3.46/3.47/1.04
3D14	1.78	-52.21	1	1.51	2.83	-60.16	3	3.07/2.10/2.25
3EBO	0.32	-34.98	1	1.15	0.18	-53.06	3	1.08/3.34/1.45
3EHY	1.76	-34.37	1	2.57	2.62	47.06	3	0.52/2.97/0.65
3F0T	0.96	-39.60	1	2.51	1.04	-27.14	3	4.04/2.66/2.46
3FDZ	2.23	9.30	1	0.67	1.99	546.54	2	3.19/3.88
3FMH	1.91	-47.03	1	0.13	1.97	-42.72	3	0.28/2.49/2.29
3FTU	0.41	-18.71	1	0.57	0.81	-3.02	1	3.80
3FUH	2.53	-9.89	1	2.55	2.20	-34.34	1	0.49
3G4K	1.67	-28.86	1	2.41	1.94	243.63	2	2.17/0.92
3GCQ	1.24	32.98	1	1.56	0.80	-50.89	1	0.25
3GQZ	1.78	620.97	1	2.27	1.94	-17.83	3	1.39/1.29/0.87

3HMM	0.22	-51.52	1	0.24	1.99	903.30	2	2.85/2.76
3HV7	0.16	-57.61	1	0.49	0.75	-66.60	2	4.56/1.11
3KBZ	1.19	-0.56	1	2.44	1.33	-54.32	2	3.28/3.86
3KCU	2.00	-33.04	1	0.97	2.33	-40.17	3	2.88/4.47/0.99
3KJD	1.23	-41.66	1	1.05	0.29	-44.98	2	4.47/0.95
3L5T	0.24	-32.54	1	0.38	0.19	-48.82	1	6.01
3LE8	5.46	-42.55	1	1.69	4.46	-10.94	3	1.39/1.67/2.83
3OZD	1.57	-51.73	1	2.31	3.19	-51.39	2	7.29/3.38
3PD9	0.39	-31.97	1	1.04	0.49	-26.73	1	2.99
3QVK	1.81	90.16	1	0.71	1.90	1.74	2	1.51/3.49
3RXJ	2.18	-4.79	1	3.02	1.81	-28.73	1	4.55
3SHC	2.00	-26.31	1	1.40	1.69	-39.78	2	1.37/1.35
3SKH	0.60	-48.52	1	0.80	0.61	-58.53	2	3.99/4.05
3SLI	3.46	-40.36	1	3.71	0.88	-65.24	2	1.89/5.75
3SMB	1.76	12.46	1	0.52	1.74	-35.83	3	4.30/2.98/0.46
3T1M	1.32	-45.54	1	2.46	1.18	-52.07	2	4.96/1.62
3UPN	2.77	-44.51	1	1.21	5.68	-59.66	2	9.62/7.54
3ZYA	0.47	-45.86	1	0.41	0.50	-16.27	3	1.00/1.33/0.79
4AQH	0.16	-10.85	1	1.53	1.89	46.27	3	1.30/8.90/4.97
4B6O	1.16	-51.02	1	2.06	1.14	-65.01	2	3.12/3.08
4B6P	2.14	-43.51	1	2.03	2.92	56.02	3	3.52/4.82/2.47
4B6R	0.21	-56.56	1	0.30	1.09	-49.90	1	0.26
4CSV	0.41	-55.72	1	1.94	3.12	120.57	2	6.80/3.44
4FU8	0.54	-29.86	1	1.94	0.91	10.69	2	1.72/5.23
4FUH	1.91	-24.02	1	2.18	2.72	990.32	3	1.58/1.52/2.51
4G8Y	1.80	-37.34	1	1.10	1.03	-38.17	2	2.85/6.21

4GJ3	2.00	-49.23	1	2.47	1.95	-55.25	3	5.73/3.89/1.21
4IFV	0.70	-29.84	1	3.28	1.36	-32.32	2	4.45/1.41
4THI	1.96	-20.80	1	0.94	2.71	-18.69	2	3.95/2.18
4WKN	0.45	-32.16	1	0.55	0.36	-41.55	1	1.17
4ZHM	2.37	16.40	1	0.32	1.89	712.60	2	4.03/1.18
5IUI	1.13	-33.63	1	1.21	2.96	-29.59	2	1.35/5.59
